# An Aging Clock Based on Immune Repertoire Features: COVID‐19 Accelerates Aging

**DOI:** 10.1111/acel.70580

**Published:** 2026-06-23

**Authors:** Xin Gao, Si‐Jia Li, Jin Li, Zi‐Hui Wang, Lv‐Tao Zeng, Ya‐Qing Ma, Ya‐Min Dang, Ying‐Min Zhang, Hong‐Lei Liu, Li‐Qun Zhang, Jing Pang, Ju Cui, Tie‐Mei Zhang, Jian‐Ping Cai

**Affiliations:** ^1^ The Key Laboratory of Geriatrics, Beijing Institute of Geriatrics Beijing Hospital, National Center of Gerontology, National Health Commission, Institute of Geriatric Medicine, Chinese Academy of Medical Sciences Beijing People's Republic of China; ^2^ Department of Clinical Laboratory Xiangya Hospital of Central South University Changsha Hunan People's Republic of China; ^3^ Department of Laboratory Medicine The First Affiliated Hospital of Xi'an Jiaotong University Xi'an Shanxi People's Republic of China; ^4^ Department of Clinical Laboratory Shandong Provincial Hospital Affiliated to Shandong First Medical University Jinan Shandong People's Republic of China; ^5^ Department of Geriatric Respiratory Disease Shandong Provincial Hospital Affiliated to Shandong First Medical University Jinan Shandong People's Republic of China; ^6^ School of Biomedical Engineering Capital Medical University Beijing People's Republic of China

**Keywords:** aging, COVID‐19, immune repertoire, machine learning

## Abstract

Aging induces immunosenescence, a progressive decline in immune function underpinning age‐related pathogen vulnerability, yet T/B cell receptor (TCR/BCR) repertoire remodeling during aging remains incompletely characterized, especially in non‐European populations. Additionally, SARS‐CoV‐2 may perturb immune homeostasis and accelerate aging, but its impact on immune repertoire aging is unclear. Here, we analyzed leukocyte DNA from 195 healthy Chinese individuals (25–93 years) and 94 post‐COVID‐19 cases via CDR3 high‐throughput sequencing. Aging correlated with shorter CDR3 sequences, reduced VDJ gene/V‐J combination diversity, declining TCR/BCR clonotype counts (179.7/507 per 3 years), expanded hyperexpanded TCR/large BCR clones, and reduced diversity (critical turning point ~60 years). These changes were intensified post‐COVID‐19, with altered amino acid usage, diminished diversity, and expanded SARS‐CoV‐2/ *Mycobacterium tuberculosis* ‐related clones. We developed a LightGBM‐based immune repertoire aging clock, validating accelerated biological aging (increased cAgeDiff) and reduced intrinsic capacity in post‐COVID‐19 individuals. Our findings reveal age‐dependent immune repertoire remodeling exacerbated by COVID‐19, deepening understanding of immunosenescence and post‐viral dysfunction, with potential clinical applications for age‐related immune decline and post‐COVID‐19 syndromes.

## Graphical abstract

We systematically profiled TCR/BCR repertoires in Chinese individuals and paired COVID‐19 samples, revealed aging‐driven remodeling and COVID‐accelerated immune senescence, and built a reliable immune repertoire aging clock for immune senescence evaluation.

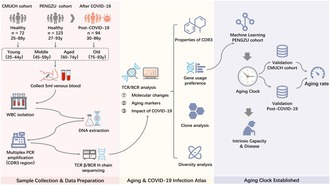

**Abbreviations**
AS: anxiety symptom
BCR: B cell receptor
cAgeDiff: Adjusted AgeDiff
CDR3: complementary determining region 3
CDR3aa: CDR3 amino acid sequence
CDR3nt: CDR3 nucleotide sequence
COVID‐19: Coronavirus Disease 2019
DS: depression symptom
GEO: gene expression omnibus
HTS: high‐throughput sequencing
LD: Levenshtein distance
MAE: mean absolute error
RMSE: root mean square error
SARS‐CoV‐2: Severe Acute Respiratory Syndrome Coronavirus 2
SHAP: SHapley Additive exPlanations
SS: stress symptom
TCR: T cell receptor
VDJ: variable (V), diversity (D), and joining (J) gene segments

## 1 Background

The immune system is a complex and dynamic network with crucial roles in maintaining physiological health and protection from various pathogens (Nikolich‐Žugich 2018). The immune repertoire, which includes highly diverse T cell receptors (TCRs) and B cell receptors (BCRs), is central to adaptive immune responses (Wu et al. 2021). TCRs and BCRs recognize and bind to specific antigens, thereby triggering an immune reaction (Parker 1993), and the ability of the immune system to respond effectively to a wide array of antigens is directly related to TCR and BCR repertoire diversity and functionality (Wu et al. 2021). At the genetic level, the diversity of TCRs and BCRs is generated through the V(D)J recombination process, which involves rearrangement of variable (V), diversity (D), and joining (J) gene segments. In the case of TCRs and BCRs, different combinations of these gene segments come together to form a unique receptor sequence (Lebedin and de la Rosa 2024). The complementary determining region 3 (CDR3) is located at the junctions of the V, D, and J gene segments in the TCRβ and BCR heavy chains and at the V‐J junction in TCRα and BCR light chains, and is particularly important in determining BCR and TCR diversity (Lebedin and de la Rosa 2024). The randomness of the V(D)J recombination process, along with the addition and deletion of nucleotides at junctions during this recombination, leads to an extremely high level of diversity in the CDR3 region, which contributes significantly to the overall diversity of TCRs and BCRs, enabling the immune system to recognize a vast number of different antigens.

In recent years, the emergence of high‐throughput sequencing (HTS) technologies has revolutionized the field of immunology by enabling comprehensive analysis of the immune repertoire. HTS allows detailed characterization of TCR and BCR sequences, providing insights into immune response diversity, clonality, and antigen specificity. The widespread application of immune repertoire sequencing has greatly advanced research into, and application of, the immune repertoire in various medical fields, including aging (Martin et al. 2015), infectious diseases (Zheng et al. 2022), autoimmune diseases (Ma et al. 2019), and oncology (Ye et al. 2018). Immune repertoire analysis can reveal the dynamic changes in the immune system during disease onset and progression, providing new perspectives for early diagnosis, treatment, and prognosis evaluation.

With increased aging of the global population, the impact of aging on the immune system has become a subject of great interest and concern. Aging is associated with a decline in immune function, a phenomenon known as immunosenescence (Brauning et al. 2022), which is accompanied by changes in the immune repertoire, such as reduced TCR and BCR diversity. For example, TCR repertoire diversity drops significantly with age, which may affect the ability of the immune system to respond to new antigens (Martin et al. 2015); however, existing knowledge in this field has several limitations. Understanding the changes that occur in the immune repertoire during aging is essential for developing strategies to enhance the immune response in older adults and improve their overall health.

Most previous studies have focused on populations of European descent, and knowledge of the immune repertoire in healthy individuals of different ages, especially in the Chinese population, remains limited (Song et al. 2022). Given the unique genetic and environmental factors that may influence the immune system in different populations, studies specifically focused on the Chinese population are necessary to gain a more comprehensive understanding of age‐related changes in the immune repertoire. Additionally, while the impact of aging on the immune repertoire is becoming clearer, the mechanisms underlying these changes are not fully understood. Factors such as genetic background, environmental exposures, and epigenetic modifications may all contribute to alterations in the immune repertoire during aging, but their relative contributions and interactions remain unclear. Here, by systematically characterizing the age‐associated immune repertoire remodeling features in a large cohort of healthy Chinese individuals with distinct genetic background and living environmental characteristics, we establish a comprehensive baseline dataset of immune repertoire aging in non‐European populations, which provides a critical foundation for subsequent studies to disentangle the relative contributions of these multi‐factorial regulators.

In the wake of the Severe Acute Respiratory Syndrome Coronavirus 2 (SARS‐CoV‐2) pandemic, an additional layer of complexity has been added to study of the immune system. Coronavirus Disease 2019 (COVID‐19) senescence, caused by SARS‐CoV‐2, has not only presented a formidable global health crisis but has also had a significant impact on human immune systems (Hu et al. 2021). COVID‐19 can lead to alterations in immune function (Diao et al. 2020; Tan et al. 2020), and there is evidence that it may even accelerate the aging process through mechanisms such as changes in DNA methylation levels (Cao et al. 2022). These findings emphasize the need to explore the intersection between aging and COVID‐19 in relation to the immune repertoire.

Most age‐related TCR/BCR studies focus on European populations; consistent aging patterns remain unclear, especially for BCR and TCR/BCR differences. Chinese population studies are small and lack full‐age baseline data (Song et al. 2022). Most COVID‐19 repertoire studies are cross‐sectional and limited to acute responses, without paired samples or links to immunosenescence, long‐term health, or age‐dependent mechanisms. No quantitative model exists for immune repertoire‐based biological age assessment. We analyzed TCR/BCR repertoires in healthy Chinese and paired pre/post‐COVID‐19 individuals, characterized aging‐ and infection‐induced remodeling, and built an immune repertoire aging clock. This work fills non‐European population gaps, reveals COVID‐19‐driven accelerated immunosenescence, and provides insights for age‐related immune decline and long COVID interventions.

## 2 Methods

### 2.1 Study Participants and Cohorts

This study included 195 healthy volunteers aged between 25 and 93 years from two sources: the “PENGZU cohort” (*n* = 123), recruited at Beijing Hospital between November 2019 and February 2020, and the “CMUCH cohort” (*n* = 72), recruited at Beijing Chest Hospital from November 2019 to October 2020. The inclusion criteria for volunteers were as follows: normal white blood cell count and ultrasensitive C‐reactive protein level within the normal range; no history of any form of tumor, with relevant tumor markers, such as carcinoembryonic antigen and alpha‐fetoprotein, at normal levels; no recent occurrence of diseases including acute cerebral infarction or acute myocardial infarction; not in any form of trauma period or postoperative recovery period; no history of any autoimmune disease; not currently undergoing any form of anti‐inflammatory or anti‐allergic treatment; and no physical or mental function problems that would prevent them from completing the study. The eligibility of all volunteers was confirmed and potential confounding factors related to underlying health conditions excluded.

This study was approved by the Ethics Committee of Beijing Hospital (Approval No. 2019 BJYYEC‐054‐02), and all volunteers signed informed consent forms.

### 2.2 Follow‐Up and Sample Designation

Follow‐up samples were collected between November and December 2022 from participants in the PENGZU cohort who had previously been diagnosed with COVID‐19 (confirmed by SARS‐CoV‐2 antigen or nucleic acid testing). These follow‐up blood samples were collected during the post‐infection period as part of a longitudinal follow‐up, rather than during the acute stage of SARS‐CoV‐2 infection. However, the exact time interval between infection/diagnosis and follow‐up sampling varied among individuals due to real‐world follow‐up conditions. A strict screening process was conducted to exclude the presence of major diseases, such as tumors, acute cerebral infarction, myocardial infarction, and autoimmune disease. Ultimately, 94 individuals (aged 30–86 years) were successfully followed up. Samples collected from individuals in the PENGZU cohort at baseline (first sampling) and after COVID‐19 were designated as the Pre‐ and Post‐COVID‐19 groups (*n* = 94 each), respectively.

### 2.3 Grouping for Analysis

For subsequent analysis, PENGZU (all baseline samples) and CMUCH cohort samples were combined into a Healthy group to study the characteristics of immune repertoire changes during aging, and the Post‐COVID‐19 group from the PENGZU cohort was used to study the characteristics of immune repertoire changes after SARS‐CoV‐2 infection. All samples were grouped according to age, as follows: Young (25–44 years), Middle (45–59 years), Aged (60–74 years), and Old (75–93 years). The basic information of all volunteers is presented in Table 1.

**TABLE 1:** Basic characteristics of samples included in this study.

	PENGZU pre‐COVID‐19 (*n* = 123)	CMUCH cohort (*n* = 72)	PENGZU post‐COVID‐19 (*n* = 94)
Condition	Healthy	Healthy	COVID‐19
Sex, *n* (%)			
Male	61 (49.6%)	36 (50.0%)	45 (47.9%)
Female	62 (50.4%)	36 (50.0%)	49 (52.1%)
Age group, *n* (%)			
Young (25–44 years)	40 (32.5%)	18 (25.0%)	24 (25.5%)
Middle (45–59 years)	35 (28.5%)	18 (25.0%)	28 (29.8%)
Aged (60–74 years)	20 (16.3%)	18 (25.0%)	27 (28.7%)
Old (75–93 years)	28 (22.8%)	18 (25.0%)	15 (16.0%)
BCR seq, *n* (%)			
Yes	122 (99.2%)	72 (100%)	92 (97.9%)
No	1 (0.81%)	0 (0%)	2 (2.13%)
TCR seq, *n* (%)			
Yes	123 (100%)	72 (100%)	91 (96.8%)
No	0 (0%)	0 (0%)	3 (3.19%)

### 2.4 Venous Blood Collection

Samples of venous blood (5 mL) were collected using purple vacuum blood collection tubes containing EDTA‐K2 anticoagulant, immediately thoroughly mixed by gentle inversion, and an aliquot used for complete blood count testing. Additionally, 2 mL of venous blood was collected into red vacuum blood collection tubes without additives and used to separate serum, which was reserved for routine blood testing and analyses of biochemical indices and tumor markers, etc.

### 2.5 Leukocyte Isolation Procedure

Leukocyte isolation was commenced within 4 h of blood collection, to preserve cell viability. Anticoagulated blood samples were centrifuged at 815× *g* for 10 min at 4°C, then the 1 mL buffy coat layer, rich in leukocytes, was extracted, mixed with 3 mL of red blood cell lysis buffer (Solarbio, China), vortexed gently, and incubated on ice for 15 min to lyse red blood cells while keeping leukocytes intact. After incubation, the mixture was centrifuged again under the same conditions, the supernatant discarded, and 0.5 mL pre‐cooled RPMI 1640 medium (Thermo Fisher Scientific, USA) and 0.5 mL of 20% DMSO cryopreservation solution (Thermo Fisher Scientific, USA) added to the pellet. Cells were resuspended and transferred to cryovials, which were placed in a pre‐cooled controlled‐rate freezing container (Thermo Fisher Scientific, USA). Once frozen, cryovials were stored at −80°C for further use.

### 2.6 Leukocyte DNA Extraction

DNA was extracted from leukocyte samples (350 μL) using a KingFisher automated nucleic acid extraction instrument (Supplier), according to the manufacturer's instructions and collected in Elution Buffer in 1.5 mL tubes. Finally, DNA quantification and quality assessment were carried out to ensure no significant degradation and availability of ≥ 0.4 μg of DNA for sequencing, by checking purity and integrity using spectrophotometry and agarose gel electrophoresis.

### 2.7 Immunorepertoire DNA Library Preparation and Sequencing

The DNA regions encoding CDR3 domains of the T cell receptor beta (TRB) and immunoglobulin heavy (IGH) chains were amplified by multiplex PCR using primers designed within conserved regions of the V and J genes that flank the regions encoding the CDR3 regions of BCRs and TCRs. This design allowed for specific amplification of the CDR3 region, which was then utilized as the foundation for the construction of high‐throughput sequencing libraries.

Subsequently, amplified single‐stranded circular DNA was transformed into DNA nanoballs (DNBs) by rolling circle replication. Each DNB contained multiple copies of the DNA sequence, enhancing the signal strength for accurate detection. DNBs were then loaded into the microwells of a high‐density DNA nanochip. High‐throughput sequencing of each DNB on the chip was conducted using combinatorial probe‐anchor synthesis on the BGI BGISEQ‐500 sequencing platform.

### 2.8 Sequencing Data Processing

Raw sequencing data were processed with SOAPnuke v1.5.6 for adapter trimming and low‐quality read filtering. Clean reads were analyzed by MiXCR v3.0.13 for alignment, CDR3 extraction, and error correction. Core clones were defined by quality‐filtered sequences; low‐frequency and single‐base variants were clustered and merged. Final clones were realigned to V/D/J references to generate clonal profiles for downstream analysis.

### 2.9 Abundance Calculations

We calculated absolute and relative abundances of V/D/J, V–J, CDR3 nucleotide sequence (CDR3nt), and CDR3 amino acid sequence (CDR3aa) from filtered clone data. Absolute abundance reflects total counts of identical sequences; relative abundance was obtained by normalization to total counts within each sample. This approach enabled robust comparisons of clonal expansion, diversity, and effects of aging and COVID‐19 on the immune repertoire.

To minimize bias from sequencing depth variation, all libraries were prepared and sequenced using standardized procedures and identical sequencing parameters. Raw reads were processed using the same quality‐control and clonotype‐calling pipeline (SOAPnuke and MiXCR) across all samples. For downstream analyses, clonotype and gene usage abundances were normalized as relative frequencies within each sample. Sequencing saturation analysis further confirmed sufficient depth for repertoire quantification (Figure S1A,B).

### 2.10 CDR3 Structural Analysis

Structural analysis of CDR3 was conducted by assessment of CDR3nt and CDR3aa sequence lengths, as well as of usage rates of the 20 amino acids. CDR3aa length and hydrophobicity are generally considered to be related to immune responses (Ritmahan et al. 2020). Based on data from the IMGT database (https://www.imgt.org), the 20 amino acids in CDR3 were classified into three categories according to their properties: hydrophilic, hydrophobic, and neutral.

### 2.11 Clonal Size Analysis

Clonal size was visualized using the WeightedTreemaps package (version 0.1.1) in R. Clones were classified into five categories (Rare, Small, Medium, Large, Hyperexpanded) via the immunarch package (version 0.8.0) with default homeo parameters, enabling in‐depth analysis of clonal expansion patterns.

### 2.12 Clonal Diversity Analysis

The diversity index of the immune repertoire serves as a quantitative gauge of the variety of immune cell receptors (e.g., TCRs or BCRs) within an individual's immune system, essentially mirroring the capacity of immune cells to recognize and react to diverse antigens, including viruses, bacteria, and other foreign substances (Britanova et al. 2014; Hou et al. 2016). A higher level of immune repertoire diversity indicates that an immune system has a broader antigen recognition range and can more proficiently handle a wide array of infections.

The Shannon–Wiener index (alternatively referred to as the Shannon diversity index or Shannon entropy) is used to measure species diversity in ecosystems (Hill 1973); it takes into account two crucial aspects of biodiversity, species richness and evenness, and is calculated using the following formula:

$$H^{\prime }=-\sum_{i=1}^{S}P_{i}\;\ln\left(P_{i}\right)$$

where H′ represents the Shannon–Wiener index, *S* is the total number of distinct clonotypes in the sample, and *P*_*i*_ is the relative abundance of the *i*th clonotype, which is simply the proportion of that particular type within the sample. The merit of this index is that it provides a well‐balanced assessment of diversity by considering both clone richness and evenness.

The Gini coefficient is commonly employed in economics to measure distribution inequality and can also be applied in the context of biodiversity to evaluate the unequal distribution of relative clone abundances. The formula for the Gini Coefficient is:

$$G=\frac{\sum_{i=1}^{n}\sum_{j=1}^{n}|P_{i}-P_{j}|}{2n\sum_{i=1}^{n}P_{i}}$$

where *n* is the total number of clones, and *P*_*i*_ and *P*_*j*_ are the relative abundances of the *i*th and *j*th clones, respectively. The value of the Gini coefficient ranges from 0 to 1; a value closer to 0 implies a more equal distribution (meaning each clone has a similar relative abundance), while a value closer to 1 indicates a highly unequal distribution (suggesting that all the abundances are concentrated in just one clone). In immune repertoire analysis, a lower Gini coefficient indicates a more even clonal distribution, while a higher coefficient suggests dominance by a few clones. This index intuitively reflects clonal inequality and reveals immune diversity and antigen response specificity.

### 2.13 Clonal Sequence Similarity Analysis

Levenshtein distance (LD) is a metric used to quantify the minimum number of single‐character edits (deletions, insertions, or substitutions) required to change one string into another (Zhang et al. 2020). We used LD to evaluate CDR3aa similarity among the top 1000 clones per sample, calculated with the R stringdist package (version 0.9.10). Focusing on the top 1000 clones standardized analysis across samples, reduced bias, and lowered computation load.

Clones were defined as being similar if they were part of a cluster where the pairwise LD was ≤ 1; this criterion allowed for a maximum of one amino acid change between pairs of CDR3 clone sequences (Zhang et al. 2020). After LD calculation, clustering and network visualization were performed with the R igraph package (version 1.3.5). Cluster numbers were counted to reflect clonal similarity. In the network, nodes represent clones; node size corresponds to connectivity, and colors are random. This visualization intuitively displays clonal similarity patterns in the immune repertoire.

### 2.14 Clonal Annotation and Functional Analysis

TCR clone annotation databases are well‐developed, but BCR databases remain scarce; thus, only TCR clones were annotated in this study. We downloaded CDR3aa sequences and antigen annotations from VDJdb (Goncharov et al. 2022), IEDB (Vita et al. 2019), and McPAS‐TCR (Tickotsky et al. 2017), integrated and deduplicated the data to obtain 182,283 unique functional TCR CDR3aa sequences, then matched our sequencing data against this reference to identify corresponding antigens.

To explore overlaps between peripheral blood–expanded TCR clones and tumor‐infiltrating clones, we obtained TRB sequences from the GEO database (GSE139555 and GSE149652), which include multiple solid tumors and adjacent normal tissues. We intersected the top 1000 expanded peripheral blood clones with tumor and paracancerous sequences and counted shared clones, revealing potential links between circulating TCR clones and the tumor microenvironment.

### 2.15 Analysis of Clone Overlap Among Individuals

It has been hypothesized that individuals may possess immune system clones that have the potential to be directed against common antigens and these clones are considered to have crucial roles in pathogen‐specific responses and combating infections (Hou et al. 2021). Nevertheless, a prominent characteristic of TCR and BCR repertoires is the high level of heterogeneity among individuals. Hence, individuals typically only share a relatively small number of clones (Xu et al. 2023). To quantitatively assess the similarity in clone composition between different samples, the R immunarch package (version 0.8.0) was employed to calculate the overlap coefficient values of TCR and BCR clones (Hou et al. 2021). The overlap coefficient is a standardized metric of overlap similarity, computed by dividing the number of shared clones (intersections) by the size of the smaller of the two sets being compared, allowing objective measurement of the degree of clone sharing among individuals and providing insights into the similarities and differences of their immune repertoires.

### 2.16 Aging Clock and Aging Rate

The training (PENGZU cohort), test (CMUCH cohort), and COVID‐19 infection (Post‐COVID‐19) datasets were used to evaluate aging clock and aging rate. Our study identified consistent age‐dependent TCR/BCR repertoire remodeling, including altered CDR3 features, reduced clonal diversity, disrupted clonal homeostasis, and stable V(D)J/V–J usage changes. These age‐associated signatures serve as ideal biomarkers for biological aging. We thus built a machine learning–based immune repertoire aging clock using TCR/BCR V/D/J/V–J usage rates as features (instead of global gene expression). The model was evaluated in the training (PENGZU), test (CMUCH), and post‐COVID‐19 datasets to assess aging clock performance and aging rate. The actual age of participants at the moment of sample collection was designated as the target variable. For the training dataset, the usage rates of all TCR and BCR V, D, J, and V–J gene combinations served as features within the aging prediction model.

Aging clock models were constructed using the tidymodels package (version 1.2.0) with nine distinct algorithms: Random Forest, Decision Tree, XGBoost, Lasso, Support Vector Machine (SVM), Neural Networks, Linear Regression, LightGBM, and K‐Nearest Neighbor (KNN). Subsequently, mean absolute error (MAE) values were compared within an external test set (CMUCH cohort). The algorithm that demonstrated the best performance was then chosen to formulate the final aging clock model.

Once the final aging clock was established, the difference between the predicted and actual age (AgeDiff), which served as a measure of the aging rate of individuals, was calculated using the following formula (Han 2024; Moqri et al. 2023; Zhu, Chen, et al. 2023):

AgeDiff=PredictedAge–Age

This parameter provides insights into impacts on health, disease, and longevity; a positive value signifies accelerated aging, whereas a negative value signifies a slower aging rate (Han 2024; Moqri et al. 2023; Zhu, Chen, et al. 2023). To isolate the influence of actual age on AgeDiff and align with published methods (Zhu, Chen, et al. 2023), AgeDiff was adjusted using a polynomial model fitted to age:

$$\text{cAgeDiff}=\text{AgeDiff}–\text{loess}\;\left(\text{AgeDiff}\sim \mathrm{Age}\right)$$

Adjusted AgeDiff (cAgeDiff) was then used as the ultimate metric for evaluating the aging rate of individuals.

To evaluate the variation in aging rates within each cohort and account for differences in cohort size, the PENGZU cohort was classified into three groups based on cAgeDiff quartiles (Q1, Q2, Q3) from the training dataset, as follows: quick‐aging (cAgeDiff > Q3), average‐aging (Q1 ≤ cAgeDiff ≤ Q3), and slow‐aging (cAgeDiff < Q1). Subsequently, the proportions of these different aging rate groups within each cohort (PENGZU, CMUCH, and Post‐COVID‐19) were computed and compared using chi‐square tests to facilitate detailed analysis of the aging patterns and differences among the cohorts, and identify factors potentially contributing to variations in aging rates.

### 2.17 Sample Size Saturation Analysis

Saturation analysis was conducted to evaluate whether sample size was adequate. The process was initiated using 20 samples. In each iteration, one additional sample was randomly selected and incorporated into the training dataset. After repeating this process 100 times for each sample size, the average values of “MAE” (Mean Absolute Error), “RMSE” (Root Mean Square Error), and “Spearman” correlation coefficient were calculated. This iterative procedure was designed to identify the point at which further increasing the sample size would no longer lead to a significant improvement in the model's performance.

### 2.18 Measurement of Intrinsic Capacity

The calculation of intrinsic capacity in this study involved multiple steps. First, a psychological score was calculated by summing depression symptom (DS), anxiety symptom (AS), and stress symptom (SS) scores, along with weighted scores for life self‐assessment and health self‐assessment, all obtained using questionnaires, according to the formula:

Psychological=DAS+Life×2.5+Health×20+1

Second, scores were obtained based on specific cognitive, hearing, BMI, grip strength (right hand for men and left hand for women), and walking speed ranges and the vitality score calculated as follows:

Vitality=BMI/5+4*Grip strength/5.

Third, weights for each dimension (cognitive, hearing, vitality, walking speed, and psychological) were calculated using the entropy weight method.

Fourth, intrinsic function scores were obtained by weighted summation of the sub‐dimensions, followed by a Box‐Cox transformation with a positive *λ* to normalize the data distribution. Finally, scores were standardized from 0–1 and linearly mapped to 0–100.

### 2.19 Statistical Analysis

All statistical analyses and data visualizations were conducted using R software (version 4.1.3). The Wilcoxon rank‐sum test was employed to compare differences between two groups, and the Kruskal–Wallis test was used for comparisons of multiple groups. Spearman's rank correlation was applied for correlation analysis. These statistical methods are non‐parametric tests, which do not rely on the assumption of normally distributed data. A *p*‐value < 0.05 was considered to indicate statistical significance.

## 3 Results

### 3.1 Age‐Related Variations in TCR and BCR Nucleotide and Amino Acid Characteristics

TCR and BCR CDR3 sequence data were generated from 195 healthy volunteers aged 25–93 years, including the PENGZU (*n* = 122) and CMUCH (*n* = 72) cohorts, recruited from November 2019 to February 2020 in two separate hospitals (Table 1).

**FIGURE 1:**
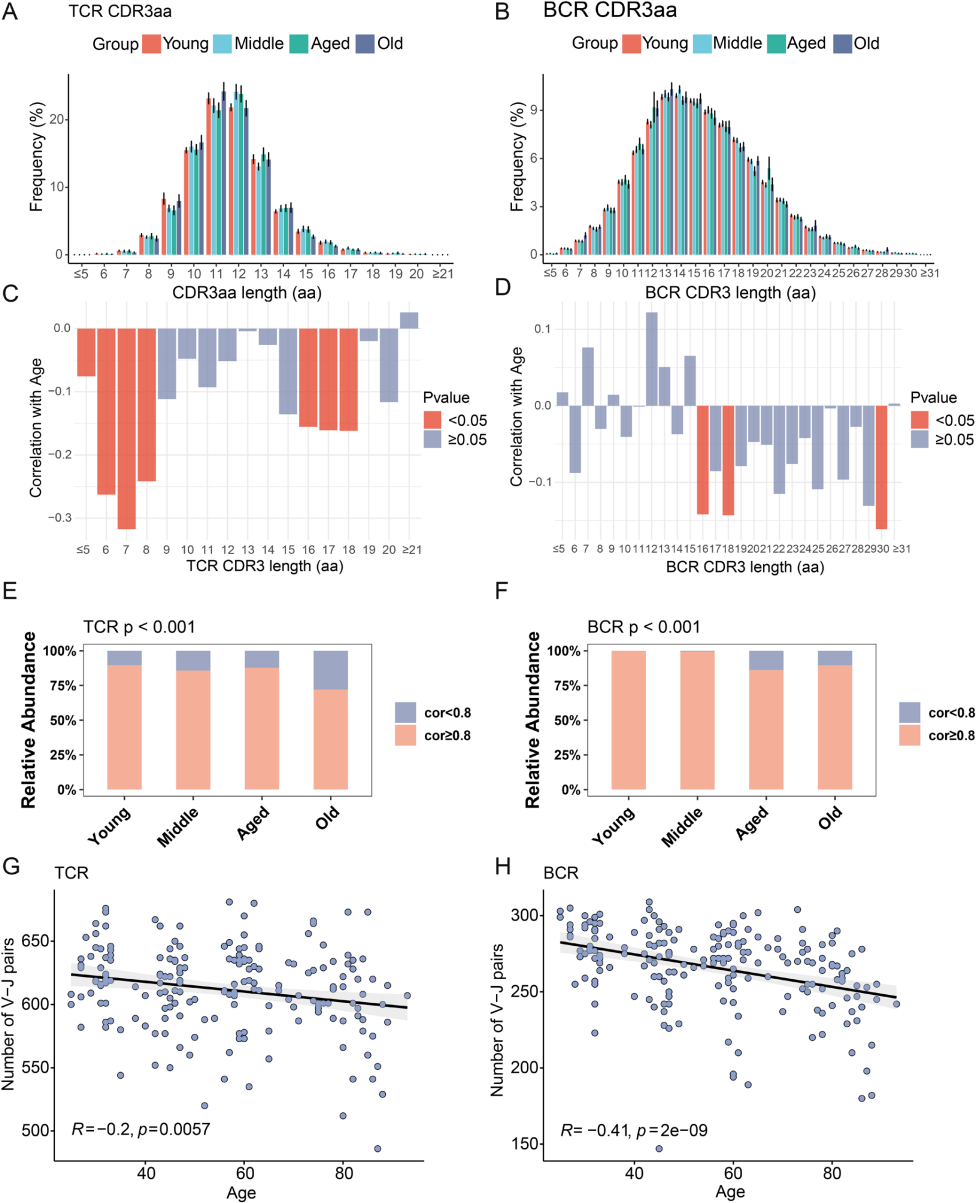
Relationships between CDR3aa and V–J gene combination types according to age. (A, B) Distributions of TCR and BCR CDR3aa lengths. (C, D) Correlation analysis of TCR and BCR CDR3aa length frequency with age. (E, F) Similarity analysis of CDR3aa length distributions among individuals. Using similarity coefficient (cor) 0.8 as the cutoff value, the proportions of sample pairs with cor values ≥ 0.8 and < 0.8 were calculated according to age group and compared by chi‐square test. (G, H) Correlations of numbers of V–J gene combination types with age.

TCR CDR3 nucleotide (CDR3nt) sequence lengths were mainly distributed in the range 31–36 bp (Figure S2A), while BCR CDR3nt lengths were primarily in the range 37–42 bp (Figure S2B). We calculated Spearman correlations for each individual CDR3 length bin independently. With aging, the immune repertoire undergoes global contraction: most frequent/functional length bins decrease, while only a few rare bins show small increases. Thus, most bins show negative correlations with age. Analysis of correlations with age demonstrated that TCR CDR3nt sequence lengths were mainly negatively correlated with age (Figure S2C,D). The functional region derived from CDR3nt, comprising the CDR3 amino acid (CDR3aa) sequence, is directly involved in antigen recognition. The most common TCR and BCR CDR3aa lengths were 11–12 and 13–14 aa, respectively (Figure 1A,B) and these were also generally negatively correlated with age (Figure 1C,D).

We further explored the differences among individuals in TCR and BCR CDR3aa sequence length distributions by conducting Spearman's correlation analysis to compare the CDR3aa sequence length distribution frequencies across various samples and examining the proportion of specific similarity results according to age group using a coefficient cutoff value of 0.8. Samples were classified into Young (25–44 years), Middle (45–59 years), Aged (60–74 years), and Old (75–93 years) age groups, and the proportions of sample pairs among groups with similarity coefficients ≥ 0.8 and < 0.8 were compared using the chi‐square test (Mai et al. 2023). As age increased, there was a significant decline in CDR3aa sequence length distribution similarity among samples (Figure 1E,F), indicating a loss of homogeneity of the immune repertoire. This result indicates that during age‐related immunosenescence, the CDR3 length distribution undergoes substantial remodeling, and the divergence of immune repertoire sequence profiles across individuals increases with age.

The frequencies of different types of CDR3aa in the entire population are presented in Figure S3A,B. Analysis of correlations with age demonstrated that TCR CDR3aa frequency exhibited more pronounced age‐related changes than that of BCR (Figure S3C).

### 3.2 Age‐Associated Alterations in VDJ and V–J Gene Combination Usage

The usage rates of all VDJ genes and V–J combinations are visualized in Figure S4A–H. Genes with potentially dominant roles in the immune response are presented in Figure S5A–F. Each V/D/J gene and each V–J combination was tested by independent Spearman correlation. Due to age‐related repertoire contraction, most dominant V genes and common V–J combinations decline, while only a few rare genes increase slightly. This results in predominantly negative correlations. Analysis of correlations with age demonstrated that VDJ gene usage rates were mainly negatively correlated with age (Figure S5G–L). Similarly, V–J combination usage rates were also predominantly negatively correlated with age (Figure S6A,B). Moreover, the number of V–J combination types decreased notably with age (Figure 1G,H), while similarity analysis of VDJ gene usage rates among samples demonstrated that there were greater differences among samples with increasing age (Figure S7A–D).

### 3.3 Age‐Related Patterns in TCR/BCR Clone Characteristics and Their Implications

**FIGURE 2:**
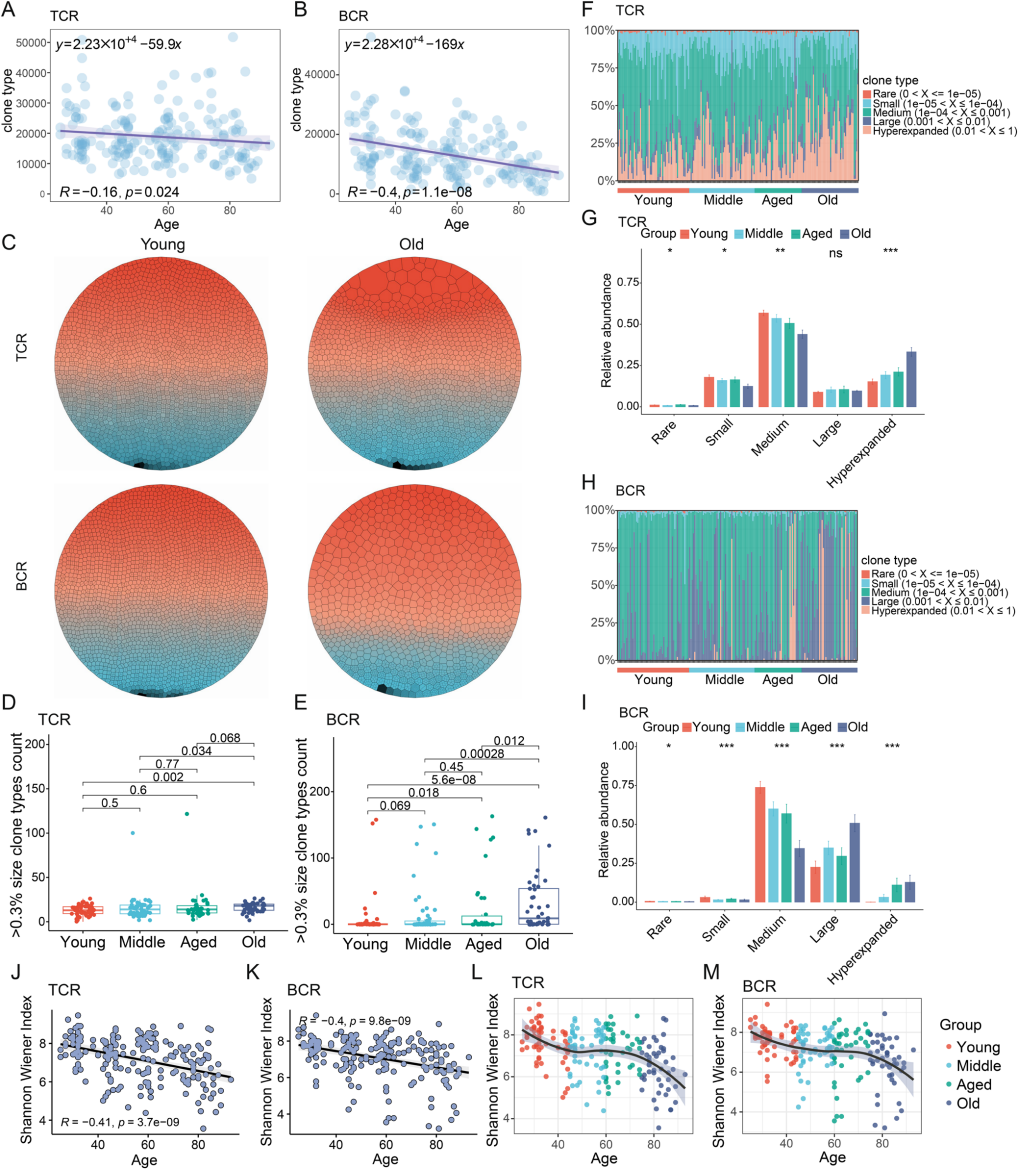
Characteristics of clone changes during aging. (A, B) Changes in TCR and BCR clonotype counts with age. (C) Clone sizes of representative samples from young and old individuals. In the Voronoi treemap, each polygon represents a unique CDR3aa clonotype and its area is proportional to its relative frequency within the sample. The color gradient (from blue to red) indicates clone abundance, with redder‐colored polygons representing higher‐frequency clonotypes and bluer polygons representing lower‐frequency clonotypes. (D, E) Comparison of clonotype counts > 0.3% in each age group. (F, G) TCR clonotype proportion homeostasis analysis. *p < 0.05, **p < 0.01, ***p < 0.001, ns, no significant. (H, I) BCR clonotype proportion homeostasis analysis. **p < 0.01, ***p < 0.001. (J, K) Analysis of correlations between clone diversity (Shannon–Wiener index) and age. (L, M) Locally weighted regression (LOESS) analysis of clone diversity and age.

Clonotype count, which denotes the number of specific clonotypes in an immune repertoire, was analyzed separately for TCRs and BCRs. Both TCR and BCR clonotype counts declined significantly with age (*p* < 0.05); the decline rate for TCR clonotype count was 179.70 per 3 years and that for BCR was 507 per 3 years, indicating a more rapid decrease in BCR clonotype count (Figure 2A,B).

Clonotype proportion (size) is vital to understanding immune responses and aging. Clonotype sizes in various samples are visualized in Figure 2C, and show that individual clonotypes tended to enlarge with age (Figure 2D,E), suggesting that aging impacts immune function by decreasing clonotype counts and increasing their proportions.

For a more detailed analysis of TCR and BCR clonotype size changes with age, we evaluated clonotype size homeostasis, with clonotypes categorized as rare, small, medium, large, and hyperexpanded (Zhu, Chelysheva, et al. 2023). For TCR (Figure 2F,G), the proportion of hyperexpanded clonotypes increased significantly with age, while those of small and medium clonotypes decreased (*p* < 0.01), and large clonotypes showed no significant change (*p* > 0.05), while the increased proportion of hyperexpanded clonotypes was prominent in the Old participant group (*p* < 0.001). For BCR (Figure 2H,I), large clonotypes showed a clear increasing trend with age, followed by hyperexpanded clonotypes, while the proportions of small and medium clonotypes decreased significantly (*p* < 0.001). In older age participants, TCRs included more hyperexpanded clonotypes, while BCRs were characterized by an increase in large clonotypes.

It has been hypothesized that individuals may carry TCR/BCR clones against common antigens, which are crucial for pathogen responses (Hou et al. 2021); however, TCR/BCR repertoires are highly heterogeneous, with few shared clones (Xu et al. 2023). Next, we calculated the overlap coefficient of TCR/BCR clones among samples and found that most samples from different age groups had low overlap coefficients (Figure S8A,B). After dividing results by the cutoff value (0.2), an increased overlap coefficient was detected among Old group samples (chi‐square test) (Figure S8C,D).

As we were unable to detect clones shared among all samples, intersection analysis was conducted using samples grouped by age (Figure S8E,F). We identified 13,231 shared TCR clones (0.5% of total), along with 3094 shared BCR clones (0.1% of total), demonstrating that shared clones comprised small fractions of both types of receptor. Further, unique TCR/BCR clones decreased with age, implying reduced immune system ability to generate new antigen‐targeting clones.

Clone diversity is indicative of immune function (Britanova et al. 2014; Hou et al. 2016), and we found that TCR/BCR diversity decreased with age (Figure 2J,K), while clone abundance became more uneven (Figure S8G,H). Nonlinear fitting suggested a turning point around 60 years for changes in TCR/BCR diversity (Figure 2L,M).

### 3.4 Analysis of the Top 1000 Clones and Their Characteristics

After determining the clone proportions in each sample, we selected the top 1000 for in‐depth analysis, to highlight the most highly representative and biologically significant clones (Jin et al. 2018). Notably, the total proportions of clones in the top 1000 increased with age for both TCR and BCR (Figure S9A,B).

To further explore the characteristics of the selected clones, we calculated the Levenshtein distance between the top 1000 CDR3aa sequence clones (*n* = 195,000 sequences); the process involved in this calculation is illustrated in Figure S9C. Clusters were defined as those containing clones with a pairwise distance ≤ 1, signifying a maximum of one amino acid difference between CDR3aa sequences (Zhang et al. 2020). Interestingly, the number of TCR clusters was not significantly correlated with age (Figure S9D), whereas the number of BCR clusters increased significantly with age (Figure S9E).

In an attempt to understand the functions of these clones, we next annotated them using 182,283 known functional TCR CDR3aa sequences from relevant databases. Of the top 1000 TCR clones, 5534 were successfully annotated with antigen types. Subsequently, we examined the correlations between proportions of clones corresponding to different antigen types and age, and identified a significant positive correlation with a specific tumor disease and negative correlations with various pathogenic microorganisms (Figure S9F).

Given the potential clinical relevance of our findings, we retrieved data from the GEO database (GSE139555 and GSE149652) to determine whether the TCR clones expanded in peripheral blood were also present in tumors and found that the number of shared clones was significantly higher in tumors than in adjacent tissues (Figure S9G,H).

### 3.5 Changes in CDR3 and VDJ Gene Usage After COVID‐19

During the COVID‐19 pandemic, we conducted a follow‐up study of 94 individuals from the PENGZU cohort; ultimately, TCR‐seq and BCR‐seq data were analyzed from 91 and 92 individuals, respectively (Table 1). Post‐COVID‐19, a significant decline was observed in the similarities of abundance distributions among samples, regardless of whether CDR3 length distribution or VDJ gene usage rates were analyzed (Figures S10A–F and 3A,B).

**FIGURE 3:**
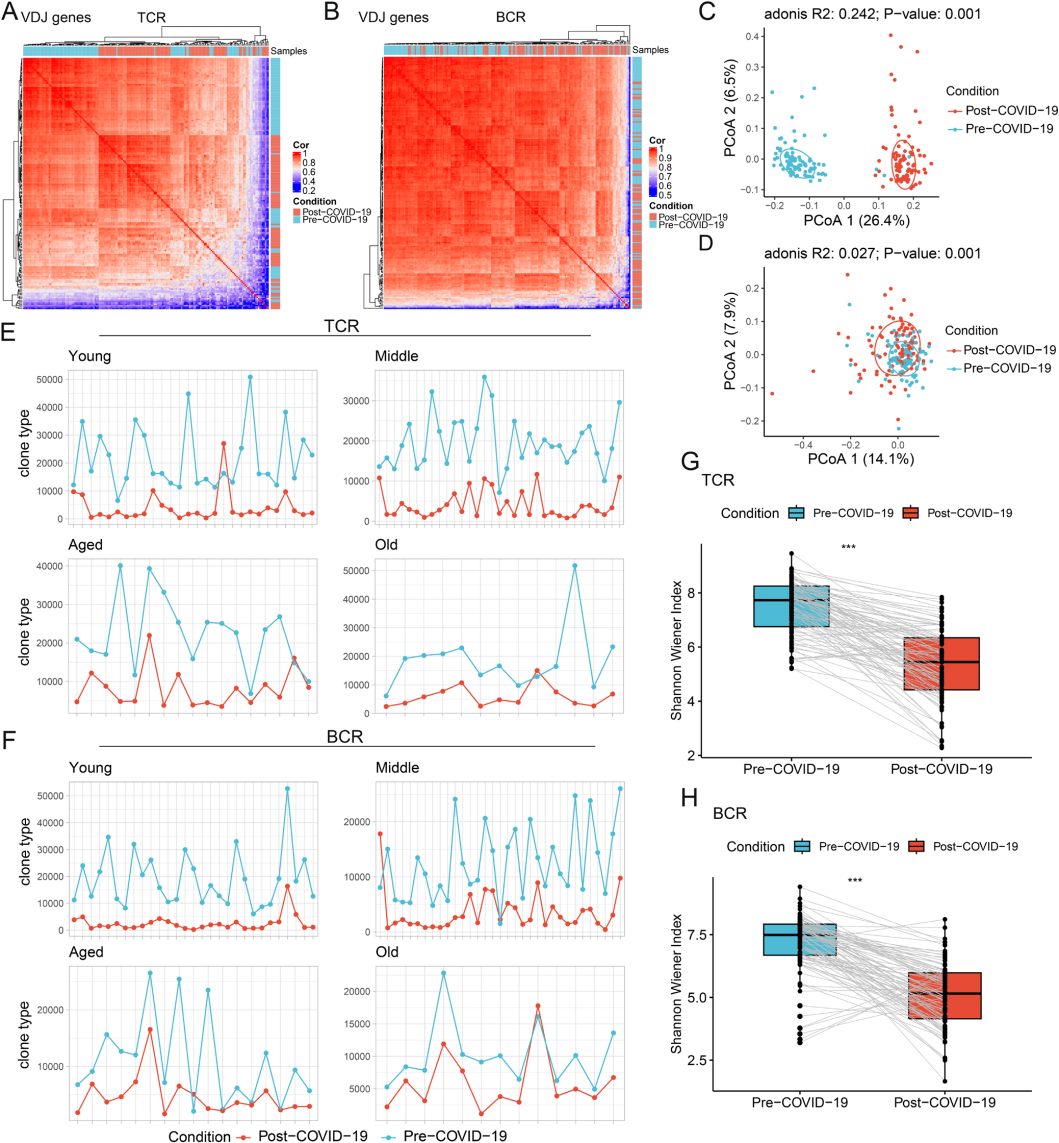
Characteristics of changes in gene usage rates and clone numbers after COVID‐19. (A, B) Heatmaps of similarity coefficient values indicating gene usage distributions among samples. (C, D) Principal component analysis of TCR and BCR gene usage rates. (E, F) Comparisons of decreases in clone numbers among different age groups after COVID‐19. (G, H) Changes in Shannon–Wiener index values after COVID‐19. ***p < 0.001.

We further conducted separate counts of the frequencies of the 20 amino acids in both TCRs and BCRs for each sample after SARS‐CoV‐2 infection and contrasted the results with those from pre‐infection samples, revealing that SARS‐CoV‐2 infection had a more pronounced influence on the amino acid usage rate in TCRs than in BCRs (Figure S11A,B).

Moreover, we found that samples could be effectively differentiated based on VDJ gene usage rates before and after SARS‐CoV‐2 infection (Figure 3C,D), suggesting a substantial alteration in gene usage rates after virus infection. These findings were based on relative‐frequency normalized data and were supported by sequencing saturation and PCA analyses, minimizing potential confounding from sequencing depth and batch effects. Our subsequent analysis of the differences in gene usage rates further corroborated this observation (Figure S12).

### 3.6 Changes in Clone Quantity and Diversity After COVID‐19

The results of shared clone analysis demonstrated that only a tiny fraction of clones were shared both prior to and after COVID‐19 (Figure S13A,B). Specifically, there were 46,557 shared TCR clones, accounting for 2.74%, and only 4211 shared BCR clones, equating to 0.32%. The low proportion of shared clones before and after COVID‐19, together with global changes in clonotype number, diversity, clonal structure, and gene usage, collectively indicates significant biological remodeling of the immune repertoire following SARS‐CoV‐2 infection. To assess the changes in shared clones within groups before and after COVID‐19, we separately calculated overlap coefficient values for both groups (Figure S13C–H). The findings indicated that the overlap coefficient between samples rose significantly after SARS‐CoV‐2 infection, suggesting induction of a similar immune response pattern. Moreover, we discovered that the number of clonotypes decreased markedly following COVID‐19 (Figure S13I,J).

To explore differences in clone responses between younger and older individuals following SARS‐CoV‐2 infection, we compared the extents of decline among TCR and BCR clonotypes in the same individuals across different age groups (Figure 3E,F), and found that the mean extent of decline reduced with increasing age (Figure S14A,B). This finding suggests that older adults are less able than younger individuals to mobilize clones focused on combating the virus after SARS‐CoV‐2 infection.

The Shannon–Wiener index represents overall immune repertoire diversity, while the Gini coefficient reflects the unevenness of clonal abundance distribution. After COVID‐19, Shannon–Wiener index values decreased significantly (Figure 3G,H), while the Gini coefficient increased significantly (Figure S14C,D), indicating that COVID‐19 resulted in a decrease in clone diversity and a more uneven distribution of clone relative abundance.

### 3.7 Clone Size and Proportion Changes Before and After COVID‐19

**FIGURE 4:**
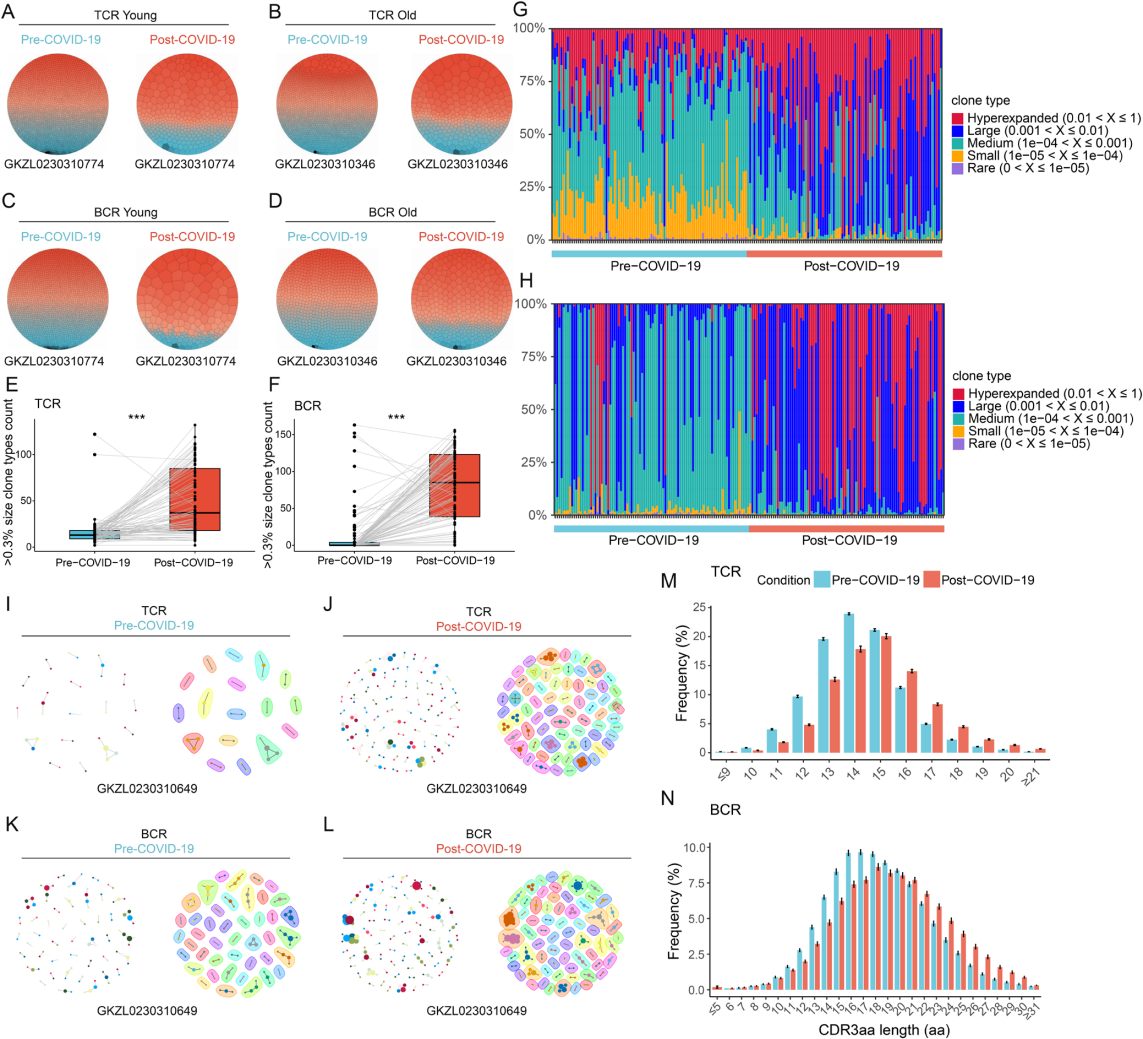
Changes in clone size and CDR3 sequences after COVID‐19. (A–D) Visualization of TCR and BCR clone sizes in the same individual before and after COVID‐19. In the Voronoi treemap, each polygon represents a unique CDR3aa clonotype and its area is proportional to its relative frequency within the sample. The color gradient (from blue to red) indicates clone abundance, with redder‐colored polygons representing higher‐frequency clonotypes and bluer polygons representing lower‐frequency clonotypes. (E, F) Comparison of numbers of TCR and BCR clones > 0.3% before and after infection. (G, H) Proportions of different sized clones. ***p < 0.001. (I–L) Cluster network diagrams of the top 1000 TCR and BCR clones from a representative sample (GKZL0230310649) with Levenshtein distance ≤ 1. In the clonotype similarity network, each node represents a CDR3aa clonotype and edges connect clonotypes with Levenshtein distance ≤ 1. Node size is proportional to node degree. Node colors are used for visualization and do not represent specific biological features. (M, N) Frequency distribution of the amino acid sequence lengths of the top 1000 amplified clones.

We also compared the alterations in clone size prior to and after SARS‐CoV‐2 infection. The changes in TCR and BCR clone sizes in representative samples of both young and older individuals before and after COVID‐19 are presented in Figure 4A–D. Visually, it is clear that the sizes of both TCR and BCR clones were significantly higher after COVID‐19, irrespective of age, and that clone size rose significantly following infection (Figure 4E,F).

Subsequently, we analyzed the proportions of rare, small, medium, large, and hyperexpanded clones in each sample; the proportions of clones of different sizes are presented in Figure 4G,H. The distribution pattern of clones of various sizes in the samples changed significantly after COVID‐19, with the proportions of hyperexpanded and large clones showing particularly notable increases. We next compared the proportions of clones of different sizes among groups (Figure S15A,B) and found that rare, small, and medium clone proportions were significantly lower in samples after COVID‐19 than those before infection, while the proportions of large and hyperexpanded clones were significantly higher (*p* < 0.001).

Moreover, we compared the clonal expansion capacity after COVID‐19 among different age groups. The proportions of rare and small TCR clones in younger patients were significantly lower than those in older individuals; however, starting from large clones, proportions were higher in younger patients, particularly for hyperexpanded clones, which were significantly more prominent (Figure S15C). The pattern in BCR clones differed somewhat from that in TCR clones. Younger patients had significantly fewer large clones than older patients, but significantly more hyperexpanded clones (Figure S15D).

### 3.8 Analysis of Expanded Clones Before and After COVID‐19

Since expanded clones may be crucial to the immune response to COVID‐19, we analyzed the top 1000 expanded clones from each sample pre‐ and post‐infection, where similar clone sequences suggest similar functions. Similarity analysis results for the top 1000 TCR and BCR clones in representative samples are shown in Figure 4I–L. TCR and BCR cluster numbers were clearly higher after infection (Figure S15E,F), reflecting the anti‐virus immune response. Further, the amino acid sequence length of the top 1000 expanded clones was significantly higher following COVID‐19 (Figures 4M,N and S15G,H).

The top 1000 pre‐ and post‐infection TCR clones were next annotated using data from known TCR clones available in databases. A total of 5642 clones were annotated with pathogen types. Among the top 6 pathogen types in these clones, SARS‐CoV‐2 was the most common (59.87% of annotated clones; *n* = 3378 clones; Figure S16A).

To identify the main pathogens targeted by these annotated clones, we calculated and compared the amplification frequency of the top 6 annotated clones in each sample. As SARS‐CoV‐2 shares epitopes with other coronaviruses, non‐infected individuals may generate cross‐reactive non‐specific clones (Loyal et al. 2021); therefore, we identified SARS‐CoV‐2‐related clones in samples from the healthy population. We noted that SARS‐CoV‐2‐specific TCRs are highly enriched in current public antigen‐specific TCR databases, which may partially contribute to the high proportion of SARS‐CoV‐2‐related clones among the top six pathogen‐associated clones. However, the significant and specific expansion of these clones in post‐COVID individuals supports their biological relevance to SARS‐CoV‐2 infection.

Comparison of amplification frequencies based on clone annotation demonstrated that the frequency of SARS‐CoV‐2‐related clones was significantly higher post‐infection (Figure S16B; *p* < 0.001). The frequency of *Mycobacterium tuberculosis* (TB)‐related clones was also increased (Figure S16E; *p* < 0.05), possibly due to SARS‐CoV‐2 activating opportunistic viral DNA to trigger latent TB infections (Can et al. 2020; Crisan‐Dabija et al. 2020; Xu et al. 2023).

### 3.9 Construction of an Immune Repertoire Aging Clock Model

The aging clock model was primarily trained using age‐associated V(D)J usage frequency features (V, D, J genes and V–J combinations), rather than CDR3 sequence‐level features. To construct an aging clock model, we selected samples with complete data for both TCR and BCR. The healthy cohort was divided into training and testing datasets, according to different sample collection locations. Specifically, 122 healthy individuals from Beijing Hospital (PENGZU cohort), all of whom had complete TCR and BCR data, served as the training dataset, while information from 72 healthy individuals from the Chest Hospital (CMUCH cohort), also with complete TCR and BCR data, was used as the testing dataset; 90 samples from the PENGZU cohort (Post‐COVID‐19) served as a disease testing dataset.

**FIGURE 5:**
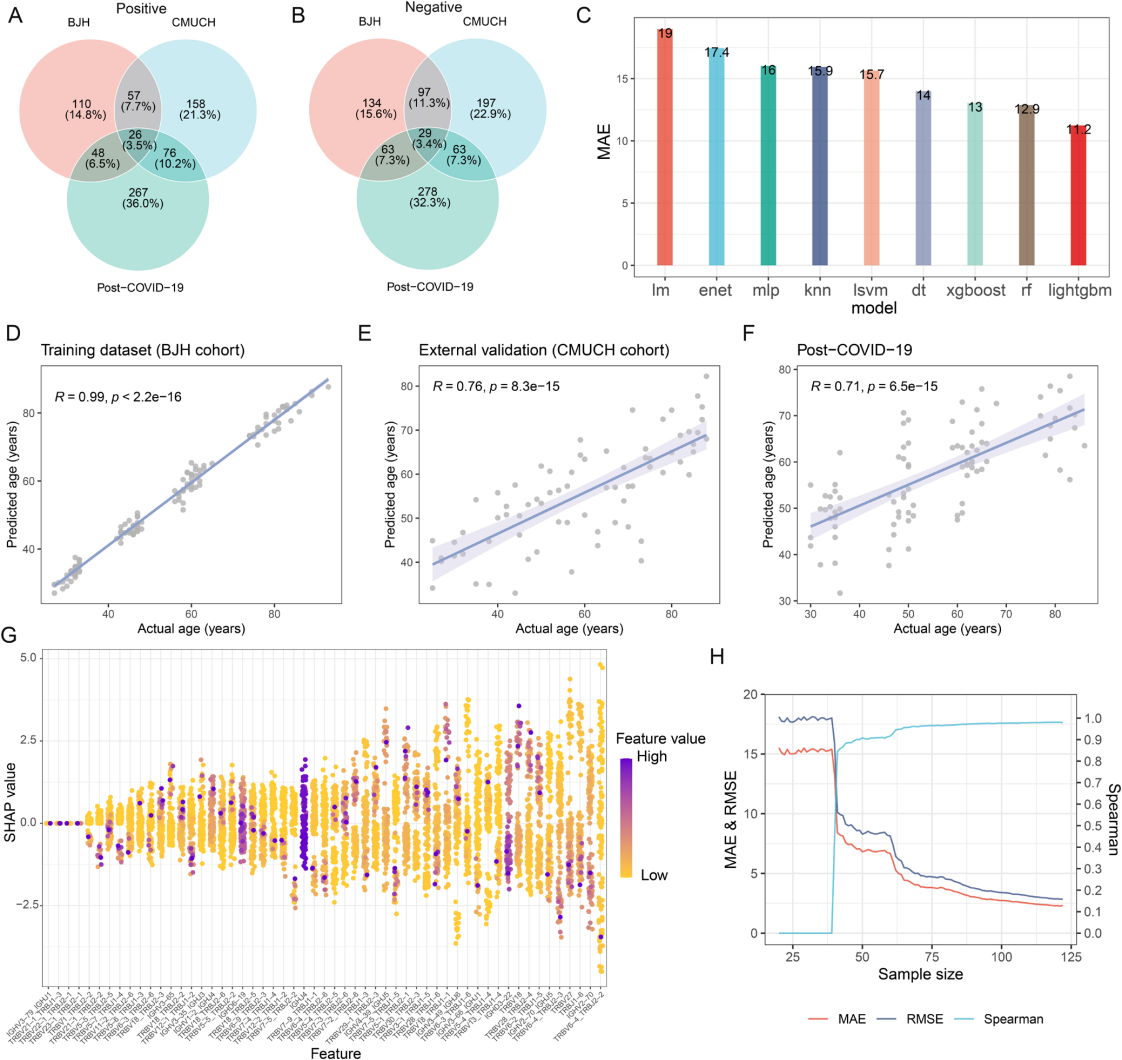
Establishment and application of an aging clock model. (A, B) Intersection of genes with consistent age‐related change trends across different cohorts. (C) Comparison of age prediction performance using 9 machine learning algorithms in an external test set. MAE, mean absolute error; lm, linear regression; enet, Lasso; mlp, neural networks; knn, K‐nearest neighbor; lsvm, support vector machine; dt, decision tree; xgboost, XGBoost; rf, random forest; lightgbm, LightGBM. (D–F) Correlation between predicted and actual age across different cohorts. (G) SHAP (SHapley Additive exPlanations) value distribution of the 55 genes. (H) Sample saturation analysis.

To improve the robustness and predictive ability of the model, we selected 55 genes that showed consistent age‐related changes in different cohorts to construct the aging clock model (Figure 5A,B). Additionally, we compared the performance of nine different machine learning algorithms using an external test set (CMUCH cohort) and found that the LightGBM model performed best (Figure 5C). The age prediction results for different cohorts are presented in Figure 5D–F. The SHapley Additive exPlanations (SHAP) value distribution for the 55 genes is presented in Figure 5G. Through sample‐size saturation analysis of the training set, we determined that the 122 reference samples used to build the clock were sufficient to achieve stable high accuracy (Figure 5H).

### 3.10 Aging Rate Changes After COVID‐19 and Their Association With Health Status

**FIGURE 6:**
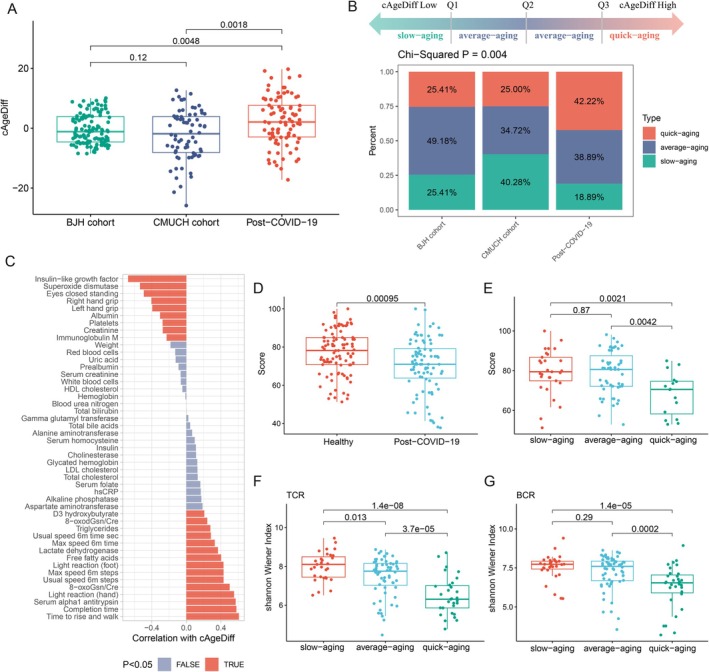
Analysis of the clinical characteristics of individuals with different aging rates. (A) Comparison of aging rates (cAgeDiff) across different cohorts. (B) Chi‐square test comparing proportions of different aging rate categories based on quartiles of cAgeDiff from the training set. (C) Analysis of correlations between aging rate and clinical laboratory test indicators. (D) Comparison of intrinsic capacity scores between healthy individuals and patients with COVID‐19. (E) Comparison of intrinsic capacity scores among individuals with different aging rates. (F, G) Comparison of TCR and BCR diversity among individuals with different aging rates.

The aging rate (cAgeDiff) computed using our aging clock increased significantly after COVID‐19 infection, yet no notable difference was detected between the healthy cohorts (Figure 6A). The proportion of individuals with accelerated aging also rose significantly following COVID‐19 infection (Figure 6B). These data imply that we successfully established an aging clock, which detected a substantial increase in the aging rate after COVID‐19 infection.

In the healthy population, to examine whether aging rate calculated using our model reflects health status, we conducted correlation analyses between the aging rate and various clinical laboratory test indicators and discovered significant correlations with indicators associated with physical function, including standing with eyes closed and grip strength of both hands, as well as blood composition indicators and insulin‐like growth factor. Remarkably, levels of superoxide dismutase in the body decreased, while oxidative nucleic acid metabolites in the urine increased significantly with increased aging rate, suggesting that aging rate has the potential to mirror health status (Figure 6C). Subsequently, we quantified intrinsic capacity scores for each participant to assess individual health status (Beard et al. 2022), revealing a significant reduction in intrinsic capacity after COVID‐19 infection (Figure 6D). Interestingly, as the aging rate accelerated, both intrinsic capacity score and immune function were significantly decreased (Figure 6E–G).

## 4 Discussion

The findings of this study provide valuable insights into the TCR and BCR changes associated with aging and COVID‐19. Our analysis of CDR3 structure revealed that CDR3 length distribution is influenced by aging, highlighting a potential impact of aging on the ability of the immune system to recognize and respond to antigens, as CDR3 length is crucial for antigen binding specificity. The differences in the most frequent CDR3aa lengths between TCRs and BCRs also suggest functional differences in the interactions of these receptors with antigens. TCRs also exhibited more significant changes in amino acid usage than BCRs, suggesting that TCR may be more vulnerable to age‐related impacts. Further, the decrease in VDJ gene and V–J combination usage with age reflects compositional contraction of the immune repertoire. Although frequencies sum to 100% per sample, aging drives global homogenization: most functional V genes and dominant V–J pairs are reduced, while only a few rare clones expand slightly. This dominant reduction leads to fewer clonotypes and lower diversity (Song et al. 2022), suggesting that the immune response may be altered with age, potentially affecting the ability to combat disease. Additionally, the faster decline in the number of BCR clonotypes, relative to that of TCR clonotypes, suggests that BCRs may be more susceptible to the effects of aging than TCRs.

Although this study did not quantify the contributions of genetics, environment, and epigenetics to immune repertoire aging, it identified key aging signatures in a large Chinese cohort and built an immune repertoire aging clock. Our population baseline data supports cross‐population comparisons to distinguish genetic and environmental effects. The 55 age‐associated genes and molecular features can serve as candidate markers. Combined with multi‐omics data, these findings will help quantify regulatory factors in immune repertoire aging. Clonal diversity reflects physiological immune capacity. The decline in clonal types with age leads to reduced immune repertoire diversity. Mika et al. found TCR repertoire diversity drops sharply before 18 years old, subsequently stabilizing until old age (Mika et al. 2023), while Britanova et al. showed that it declines linearly with age, notably by 40‐years‐old (Britanova et al. 2014); hence the rate of immune repertoire diversity decline clearly varies with age. There are no reports on BCR repertoire decline at different ages. Our data demonstrate a rapid decline in TCR and BCR diversity in the Young (25–44 years) group, which stabilized in the Middle (45–59 years) group, and accelerated again in the Aged (60–74 years) group, continuing in the Old (75–93 years) group. These findings reveal a non‐uniform rate of TCR and BCR diversity change with age, with a possible turning point around 60‐years‐old, potentially related to natural immune system aging. Furthermore, we first quantified divergent aging features of TCR and BCR. BCR clonotype count declined significantly faster (507 per 3 years) than TCR (179.7 per 3 years), confirming higher BCR sensitivity to aging. Compared with Song et al. (Song et al. 2022), our study expands analyses to multiple dimensions and provides a more comprehensive reference for immune repertoire aging. Comparison of diversity between sexes, after stratifying by age group, did not reveal any differences between men and women (Figure S17A,B), consistent with previous reports indicating that changes in immune repertoire diversity are unrelated to sex (Lian et al. 2019).

Analysis of the top 1000 expanded clonotypes showed that similarity among BCR clones increased with age, while no similar trend was observed for TCRs. This difference in aging‐related clonal changes between TCRs and BCRs has not been reported previously and warrants further investigation.

Our study also confirms the importance of TCRs in tumor immunity, as the proportion of tumor‐associated TCR clonotypes increased with age, while that of pathogen‐related clonotypes decreased. The identification of shared clonotypes between peripheral blood and various solid tumors emphasizes the potential for using peripheral blood clonotype detection to assist in tumor diagnosis and assessment of tumor immune status (Wu et al. 2020).

Our analysis of clone size and clone size distribution before and after COVID‐19 revealed differences in immune responses to COVID‐19 among different age groups, indicating that the immune systems of younger individuals may more easily generate large and hyperexpanded clones in response to COVID‐19, potentially resulting in stronger immune responses against the virus. Further, the higher CDR3aa sequence lengths detected following COVID‐19 may reflect improved clone functionality, as longer sequences can encode more functional domains to enhance protein activity and binding, thereby helping to maintain an effective anti‐virus response. It should be noted that the post‐COVID‐19 repertoire data in this study were derived from follow‐up samples collected after SARS‐CoV‐2 infection rather than during the acute infection stage. Therefore, the observed repertoire remodeling likely reflects persistent immune alterations after infection and/or the convalescent immune response. Nevertheless, because the time interval between infection and sampling was not identical for all individuals, we cannot fully distinguish acute‐phase transient effects from long‐lasting post‐infection changes. Future studies with serial sampling at defined time points (acute, early convalescence, and long‐term recovery) will be required to clarify the temporal dynamics of COVID‐19‐associated immune repertoire remodeling.

Machine learning has frequently been applied to establish aging prediction models (Haug and Drazen 2023); for example, metabolomic (Zhang et al. 2024), microbiome (Wang et al. 2024), transcriptomic (Zhu, Chen, et al. 2023), and epigenetic (Cao et al. 2022; Horvath 2013; Radak et al. 2024) aging clocks; however, immune repertoire aging clocks are lacking. The development of an aging clock based on TCR and BCR gene usage data is a significant achievement of this study. Further, our finding that COVID‐infection significantly accelerates aging, as indicated by increased cAgeDiff value, is consistent with the results of previous studies on epigenetic aging clocks (Cao et al. 2022). These data suggest that SARS‐CoV‐2 infection not only affects the immune response, but also has a profound impact on the aging process.

Aging is considered a significant factor influencing the severity of COVID‐19 (Williamson et al. 2020). Our analysis of TCR/BCR clone changes across different age groups after SARS‐CoV‐2 infection is consistent with data demonstrating that younger individuals exhibit more active immune responses (Lavezzo et al. 2020; Tabata et al. 2020), as we found a greater reduction in clone numbers and a higher abundance of hyperexpanded clones. Using paired pre‐ and post‐COVID‐19 samples, our analysis extends beyond acute immune responses to long‐term effects. The within‐subject design confirms COVID‐19 directly triggers persistent TCR/BCR remodeling rather than transient acute changes. We first reveal age‐dependent differences in post‐COVID immune responses, with younger patients displaying stronger immune plasticity. COVID‐19 enriches not only SARS‐CoV‐2‐specific clones but also *Mycobacterium tuberculosis* ‐associated clones, implying potential activation of opportunistic infection‐related repertoires. We further link post‐COVID repertoire alterations to accelerated immune aging, with elevated aging clock values associated with lower host resilience and diversity. These findings establish a direct link between COVID‐19 and accelerated immune aging, filling critical gaps in the immune mechanisms of post‐COVID conditions. This information regarding age‐related immune responses can guide clinicians in developing more personalized treatment plans.

## 5 Conclusions

In summary, this study advances immune repertoire, aging and COVID‐19 research. We established age‐stratified TCR/BCR baseline data in a large Chinese cohort, identified age 60 as a key aging turning point, and revealed divergent TCR/BCR aging patterns. We developed the first LightGBM‐based immune repertoire aging clock for precise immune aging evaluation. We demonstrate COVID‐19 accelerates immune repertoire aging and links post‐infection remodeling to host resilience and opportunistic infection risk, providing molecular targets for post‐COVID interventions. Limitations include lack of exploration on aging reversibility and interventions. Future studies may help delay age‐related immune decline and alleviate post‐COVID immune dysfunction.

## Author Contributions

**Xin Gao:** conceptualization, methodology, formal analysis, investigation, data curation, writing – original draft, visualization. **Si‐Jia Li, Jin Li, Zi‐Hui Wang, and Lv‐Tao Zeng:** conceptualization, software, writing – original draft. **Ya‐Qing Ma and Ya‐Min Dang:** conceptualization, investigation, writing – original draft. **Ying‐Min Zhang and Hong‐Lei Liu:** software, validation. **Li‐Qun Zhang and Jing Pang:** investigation. **Ju Cui:** writing – review and editing. **Tie‐Mei Zhang and Jian‐Ping Cai:** conceptualization, writing – review and editing.

## Funding

This work was supported by National High Level Hospital Clinical Research Funding (BJ‐2024‐138), CAMS Innovation Fund for Medical Sciences (No. 2021‐1‐I2M‐050), and the National Natural Science Foundation of China (No. 82170856).

## Ethics Statement

Peripheral blood samples were collected during routine procedures with written informed consent, in accordance with the approval of the Ethics Committee of Beijing Hospital (Approval No. 2019 BJYYEC‐054‐02).

## Consent

All participants signed informed consent forms and were notified about the study before participating.

## Conflicts of Interest

The authors declare no conflicts of interest.

## Supporting information


**Figure S1:** Sequencing saturation curves of TCR and BCR repertoires across all study samples grouped by age. (A) TCR repertoire saturation curve. (B) BCR repertoire saturation curve. All curves show a trend of diminishing returns in unique clone detection with increasing reads, reflecting sequencing saturation.
**Figure S2:** Distribution of TCR and BCR nucleotide segments. (A, B) Frequency distribution of TCR and BCR nucleotide segment lengths. (C, D) Analysis of correlations between the frequencies of different TCR and BCR nucleotide segments and age.
**Figure S3:** TCR and BCR amino acid usage rates. (A, B) Average amino acid frequency rankings for TCR and BCR. (C) Correlation analysis of amino acid frequencies in TRB and IGH with age.
**Figure S4:** Visualization of gene usage. (A, B) Usage rates of the top 20 TCR and BCR V genes in different age groups of each sample. (C, D) Usage rates of all TCR and BCR D genes in different age groups. (E, F) Usage rates of all TCR and BCR J genes in different age groups. (G, H) Usage rates of TCR and BCR V–J combinations in different age groups.
**Figure S5:** Analysis of TCR and BCR VDJ gene usage in immune repertoires. (A–F) Top‐ranked genes by usage rate. (G–L) Correlation analysis of TCR and BCR V gene usage rates with age.
**Figure S6:** Correlation analysis of V–J gene combinations with age. (A) TCR. (B) BCR.
**Figure S7:** Similarity analysis of gene usage rates among different samples. (A, B) Spearman similarity heatmaps of gene usage rates among different samples. (C, D) Comparisons of proportions of results with similarity coefficients ≥ 0.8 and < 0.8 across different age groups compared by chi‐square test.
**Figure S8:** Clone and diversity analysis. (A, B) Clone overlap coefficients between samples in different age groups. (C, D) Comparison of overlap coefficients in different age groups. (E, F) Intersections of TCR and BCR clones across different age groups. (G, H) Correlations between Gini coefficient and age.
**Figure S9:** Changes in and functional analysis of the top 1000 expanded clones with age. (A, B) Comparison of the total proportions of TCR and BCR clones in the top 1000 among different age groups. (C) Schematic representation of Levenshtein distance (LD) types between clone sequences (left side), with insertions, deletions, and substitutions. The upper right network diagram shows an example of clone clustering in a sample, where each point represents a clone (colors are randomly assigned). Node size correlates with node degree (degree). Connections indicate LD ≤ 1 between clones. The lower right panel depicts the network of these clones, where clusters are circled to count of the number of clusters in the sample. (D, E) Age‐related analysis of LD similarity cluster numbers. (F) Age‐related analysis of annotation proportions of the top 1000 TCR clones. (G) Comparison of shared clone numbers between the top 1000 peripheral blood TCR clones and various solid tumors and adjacent tissues in the GSE139555 dataset. (H) Comparison of shared clone numbers between the top 1000 peripheral blood TCR clones and bladder cancer and adjacent tissues in the GSE149652 dataset.
**Figure S10:** Similarity analysis of CDR3aa and VDJ gene usage rates before and after COVID‐19. (A–D) Comparison of the similarity of CDR3aa distributions between samples before and after COVID‐19. (E, F) Comparisons of the similarity of VDJ gene usage rates between samples before and after COVID‐19.
**Figure S11:** Comparison of amino acid usage rates before and after SARS‐CoV‐2 infection. (A) TCR. (B) BCR. *p < 0.05, **p < 0.01, ***p < 0.001, ns, no significant.
**Figure S12:** Differential analysis of gene usage before and after SARS‐CoV‐2 infection.
**Figure S13:** Clone analysis before and after SARS‐CoV‐2 infection. (A, B) Intersections of all TCR (A) and BCR (B) clones in samples before and after infection. (C–H) Analysis of TCR (C–E) and BCR (F–H) clone overlap coefficients between samples in groups before and after infection. (I, J) Comparison of the number of TCR (I) and BCR (J) clone types before and after infection. ***p < 0.001.
**Figure S14:** Changes in clones after SARS‐CoV‐2 infection. (A, B) The extent of decline in TCR (A) and BCR (B) clones in different age groups after SARS‐CoV‐2 infection. (C, D) Comparison of TCR (C) and BCR (D) Gini coefficient values before and after COVID‐19 infection. ***p < 0.001.
**Figure S15:** Clone and diversity analysis after COVID‐19 infection. (A, B) Analysis of TCR (A) and BCR (B) clone space steady state before and after COVID‐19 infection. (C, D) Analysis of TCR (C) and BCR (D) clone space steady state in patients of different age groups after COVID‐19 infection. (E, F) Cluster number statistics for the top 1000 TCR (E) and BCR (F) clones before and after infection. (G, H) Analysis of the amino acid sequence length of the top 1000 expanded TCR (G) and BCR (H) clones. *p < 0.05, **p < 0.01, ***p < 0.001, ns, no significant.
**Figure S16:** Functional analysis of the top 1000 amplified clones post‐COVID‐19. (A) Distribution of the top 6 pathogen types successfully annotated and the number of clones for each type. (B–G) Amplification frequency of the top 6 pathogen clonotypes before and after SARS‐CoV‐2 infection. *p < 0.05, **p < 0.01, ***p < 0.001, ns, no significant.
**Figure S17:** Comparisons of Shannon‐Wiener index values between the gender. (A) TCR. (B) BCR. ns, no significant difference; F, female; M, male; ns, no significant.

## Data Availability

Data reported in this paper will be shared by the lead contact upon request. Analysis core code is available at https://github.com/xingao0612/Core_Code. Any additional information required to reanalyze the data reported in this paper is available from the lead contact upon request.
