# Supplementary material for: An Aging Clock Based on Immune Repertoire Features: COVID‐19 Accelerates Aging
Source: Aging Cell. 2026 Jun 23;25(7):e70580. doi: 10.1111/acel.70580 (PMC13288058; doi:10.1111/acel.70580)
Supplement: Supplementary file 1 — Figure S1: Sequencing saturation curves of TCR and BCR repertoires across all study samples grouped by age. (A) TCR repertoire saturation curve. (B) BCR repertoire saturation curve. All curves show a trend of diminishing returns in unique clone detection with increasing reads, reflecting sequencing saturation. Figure S2: Distribution of TCR and BCR nucleotide segments. (A, B) Frequency distribution of TCR and BCR nucleotide segment lengths. (C, D) Analysis of correlations between the frequencies of different TCR and BCR nucleotide segments and age. Figure S3: TCR and BCR amino acid usage rates. (A, B) Average amino acid frequency rankings for TCR and BCR. (C) Correlation analysis of amino acid frequencies in TRB and IGH with age. Figure S4: Visualization of gene usage. (A, B) Usage rates of the top 20 TCR and BCR V genes in different age groups of each sample. (C, D) Usage rates of all TCR and BCR D genes in different age groups. (E, F) Usage rates of all TCR and BCR J genes in different age groups. (G, H) Usage rates of TCR and BCR V–J combinations in different age groups. Figure S5: Analysis of TCR and BCR VDJ gene usage in immune repertoires. (A–F) Top‐ranked genes by usage rate. (G–L) Correlation analysis of TCR and BCR V gene usage rates with age. Figure S6: Correlation analysis of V–J gene combinations with age. (A) TCR. (B) BCR. Figure S7: Similarity analysis of gene usage rates among different samples. (A, B) Spearman similarity heatmaps of gene usage rates among different samples. (C, D) Comparisons of proportions of results with similarity coefficients ≥ 0.8 and < 0.8 across different age groups compared by chi‐square test. Figure S8: Clone and diversity analysis. (A, B) Clone overlap coefficients between samples in different age groups. (C, D) Comparison of overlap coefficients in different age groups. (E, F) Intersections of TCR and BCR clones across different age groups. (G, H) Correlations between Gini coefficient and age. Figure S9: Changes in and fun [file ACEL-25-e70580-s001.docx]

**Supplementary Figures and Legends for**

**An aging clock based on immune repertoire features: COVID-19 accelerates aging**

Xin Gao, Si-Jia Li, Jin Li, Zi-Hui Wang, Lv-Tao Zeng, Ya-Qing Ma, Ya-Min Dang, Ying-Min Zhang, Hong-Lei Liu, Li-Qun Zhang, Jing Pang, Ju Cui, Tie-Mei Zhang^*^, Jian-Ping Cai^*^

| **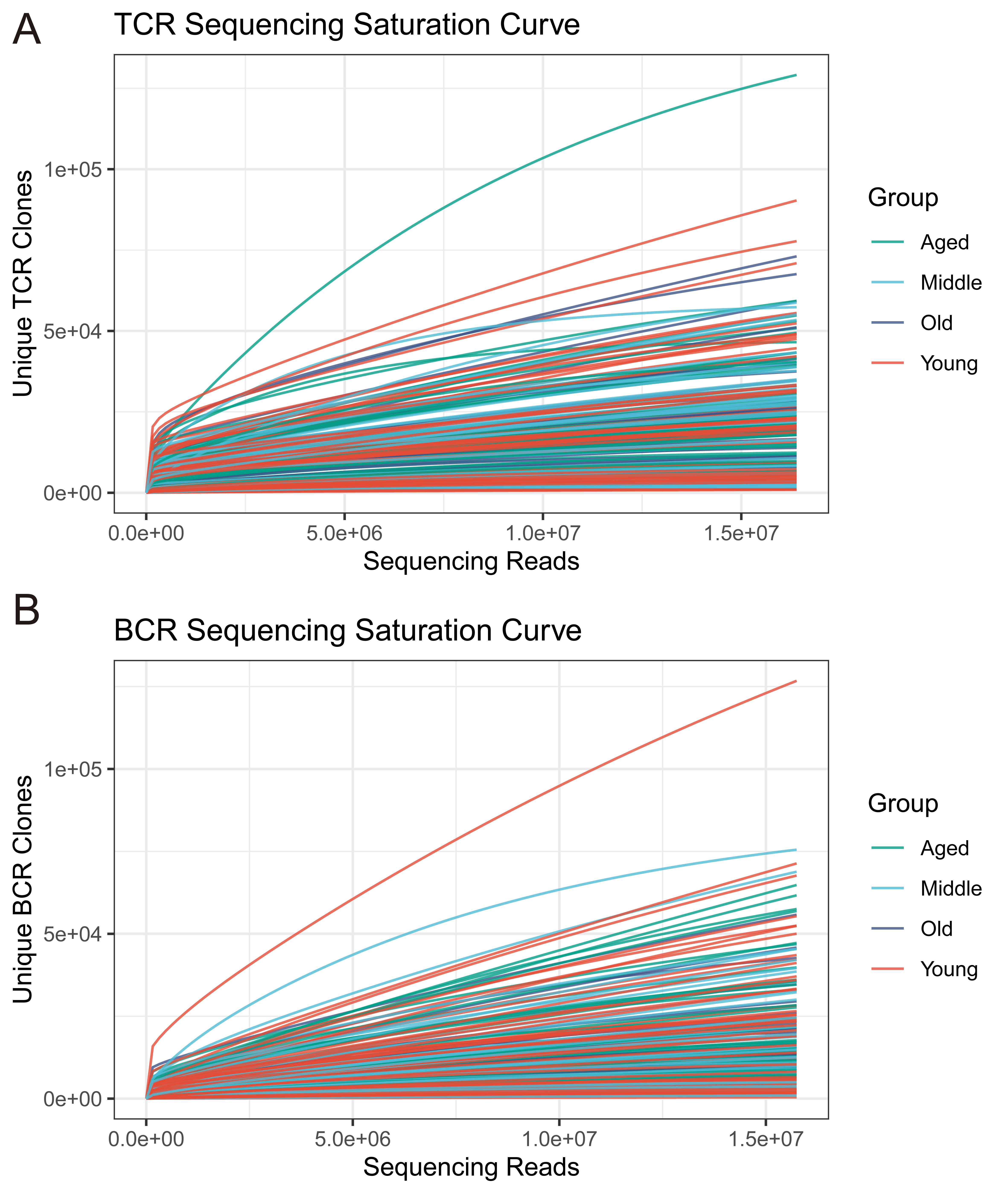Figure S1. Sequencing saturation curves of TCR and BCR repertoires across all study samples grouped by age.**  **A**,TCR repertoire saturation curve.  **B**, BCR repertoire saturation curve.  All curves show a trend of diminishing returns in unique clone detection with increasing reads, reflecting sequencing saturation. |
| --- |

| 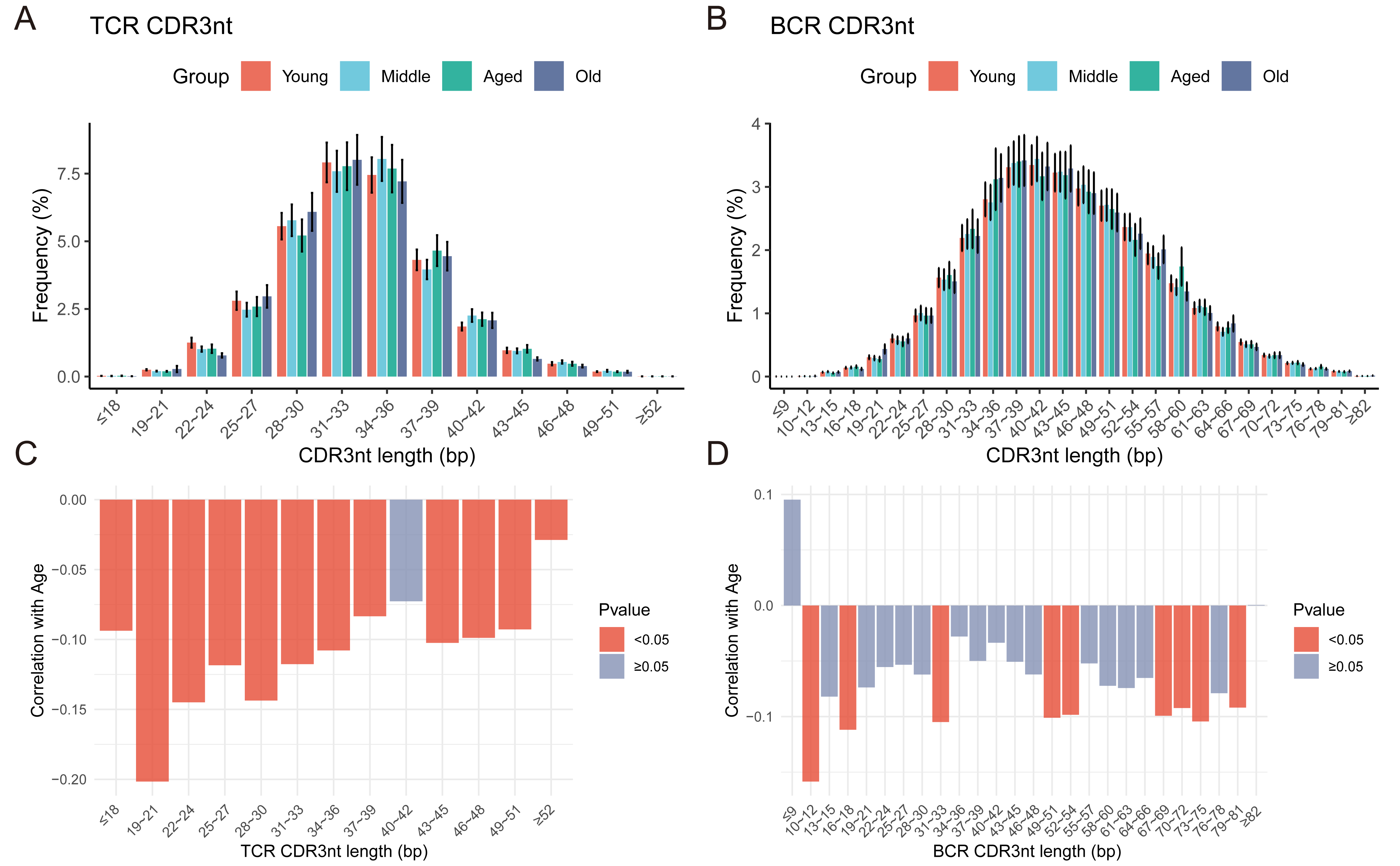 |
| --- |

**Figure S2. Distribution of TCR and BCR nucleotide segments.**

**A–B**, Frequency distribution of TCR and BCR nucleotide segment lengths. **C–D,** Analysis of correlations between the frequencies of different TCR and BCR nucleotide segments and age.

| 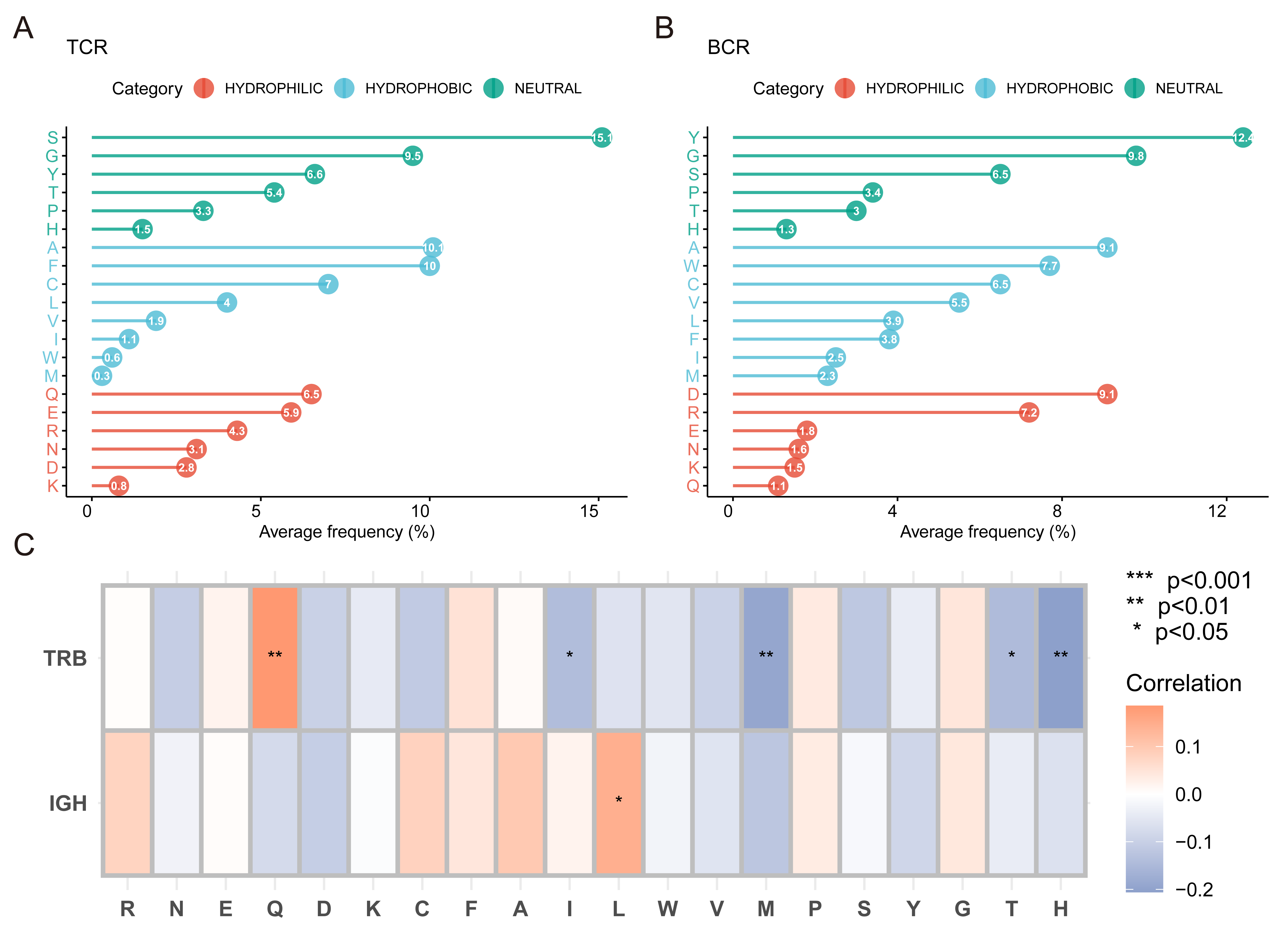 |
| --- |

**Figure S3. TCR and BCR amino acid usage rates.**

**A–B,** Average amino acid frequency rankings for TCR and BCR. **C,** Correlation analysis of amino acid frequencies in TRB and IGH with age.

**Amino Acids Classified by Polarity:**

**1. Hydrophobic**: **A**, Alanine; **C**, Cysteine; **I**, Isoleucine; **L**, Leucine; **M**, Methionine; **F**, Phenylalanine; **W**, Tryptophan; **V**, Valine.

**2. Neutral**: **G**, Glycine; **H**, Histidine; **P**, Proline; **S**, Serine; **T**, Threonine; **Y**, Tyrosine.

**3. Hydrophilic**: **R**, Arginine; **N**, Asparagine; **D**, Aspartic acid; **Q**, Glutamine; **E**, Glutamic acid; **K**, Lysine.

| 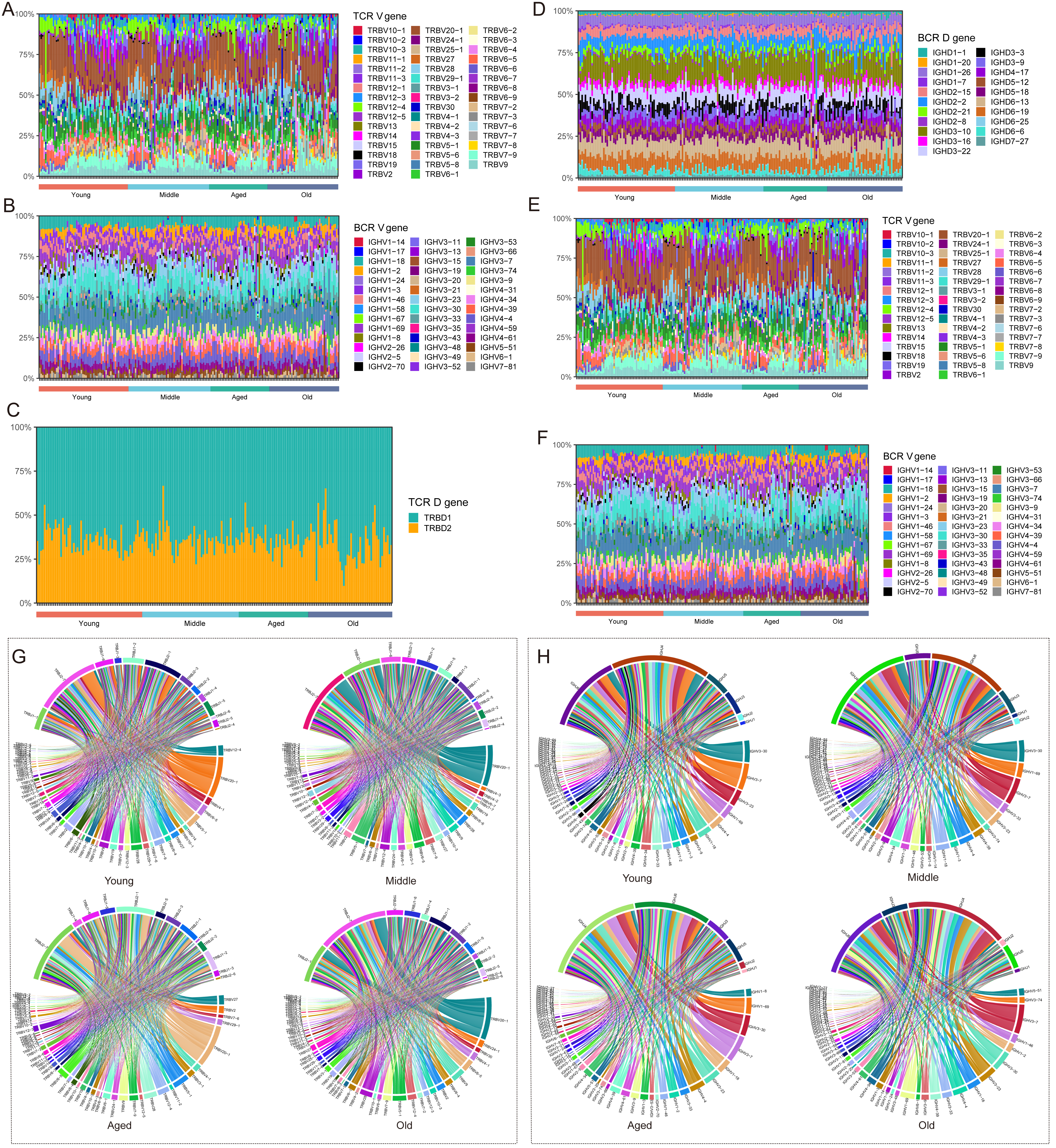 |
| --- |

**Figure S4.** **Visualization of gene usage.**

**A–B,** Usage rates of the top 20 TCR and BCR V genes in different age groups of each sample. **C–D,** Usage rates of all TCR and BCR D genes in different age groups. **E–F,** Usage rates of all TCR and BCR J genes in different age groups. **G–H,** Usage rates of TCR and BCR V–J combinations in different age groups.

| 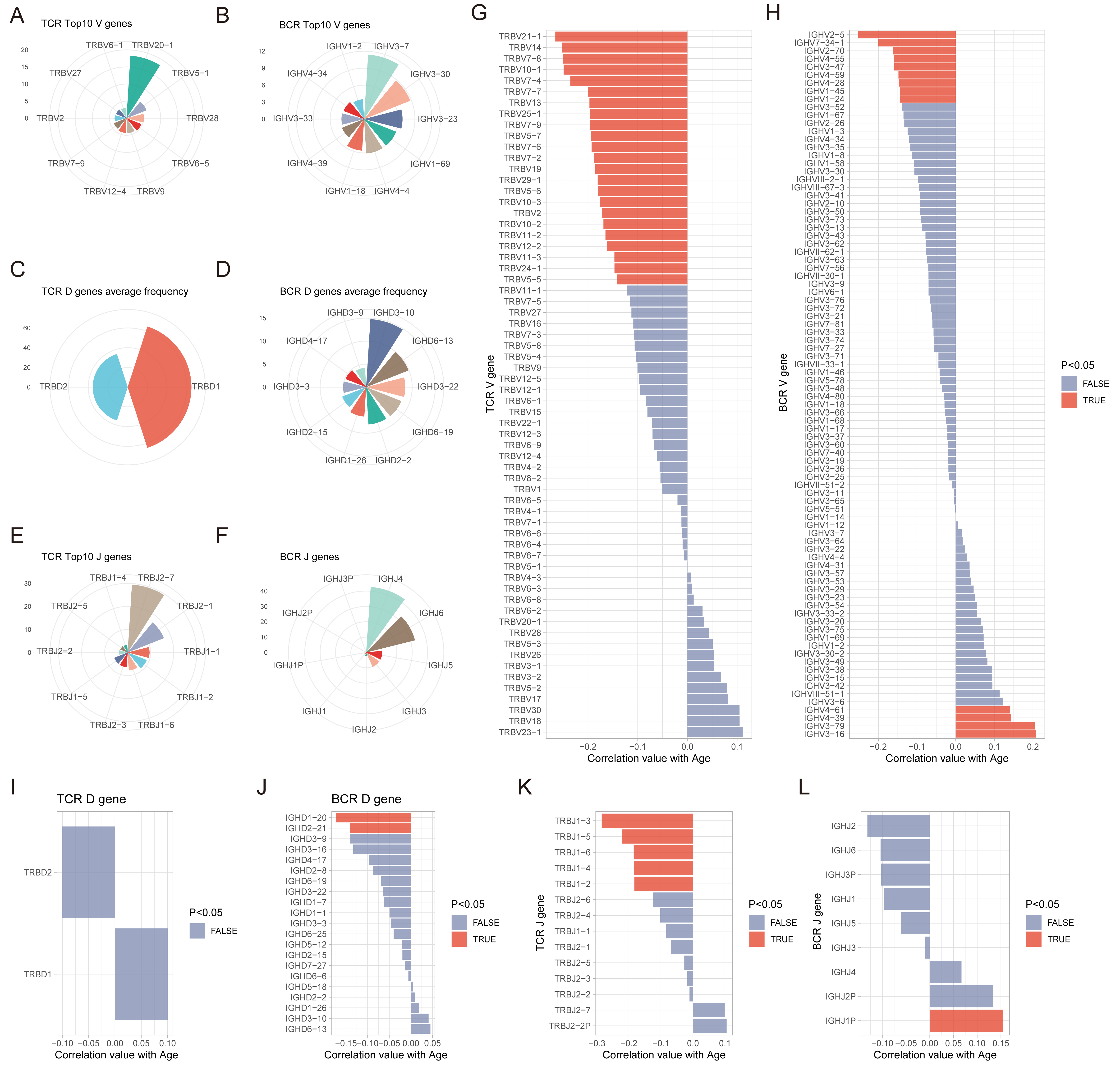 |
| --- |

**Figure S5. Analysis of TCR and BCR VDJ gene usage in immune repertoires.**

**A–F,** Top-ranked genes by usage rate. **G–L,** Correlation analysis of TCR and BCR V gene usage rates with age.

| 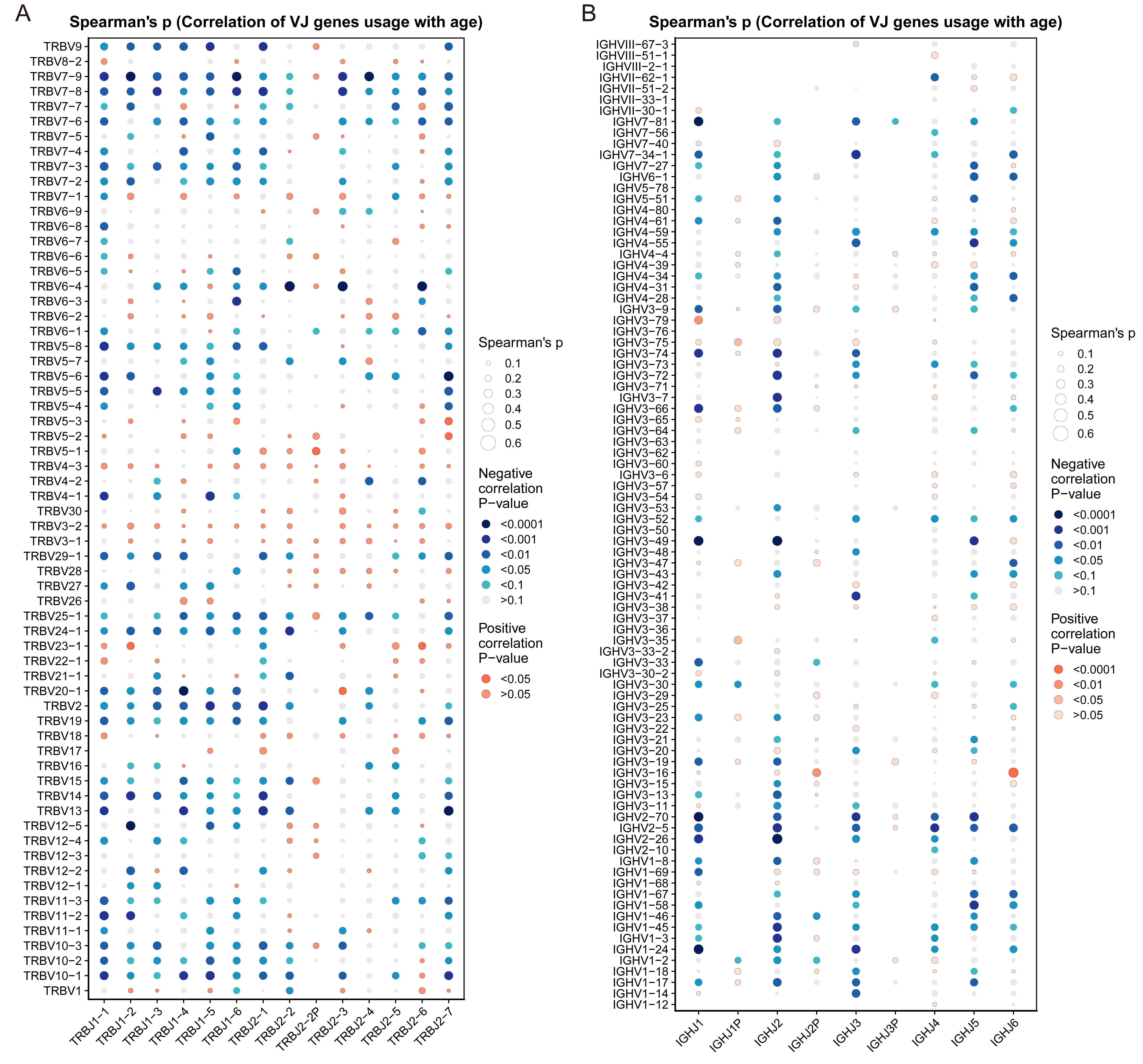 |
| --- |

**Figure S6.** **Correlation analysis of V–J gene combinations with age.**

**A,** TCR. **B,** BCR.

| 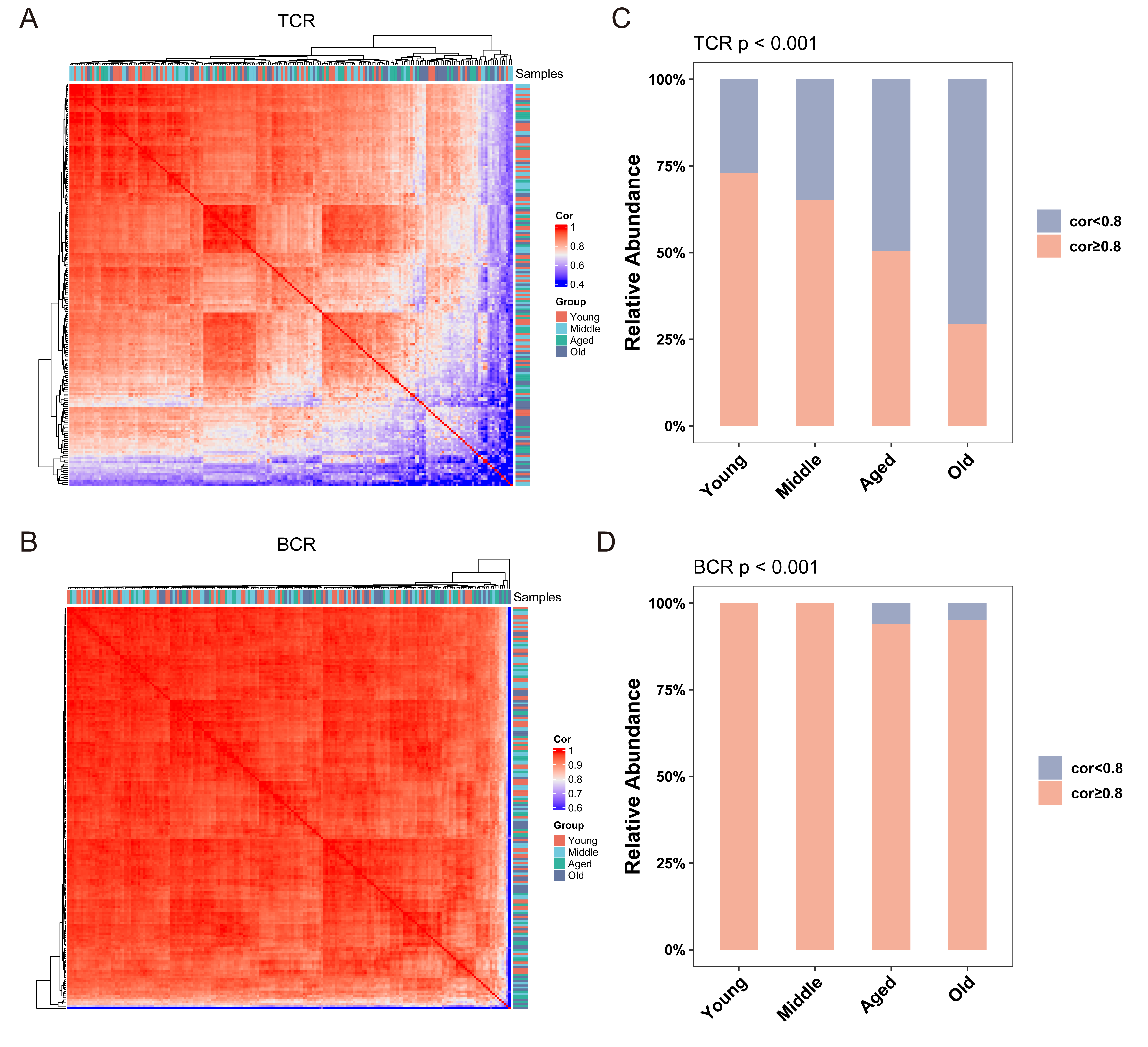 |
| --- |

**Figure S7.** **Similarity analysis of gene usage rates among different samples.**

**A–B,** Spearman similarity heatmaps of gene usage rates among different samples. **C–D,** Comparisons of proportions of results with similarity coefficients ≥ 0.8 and < 0.8 across different age groups compared by chi-square test.

| 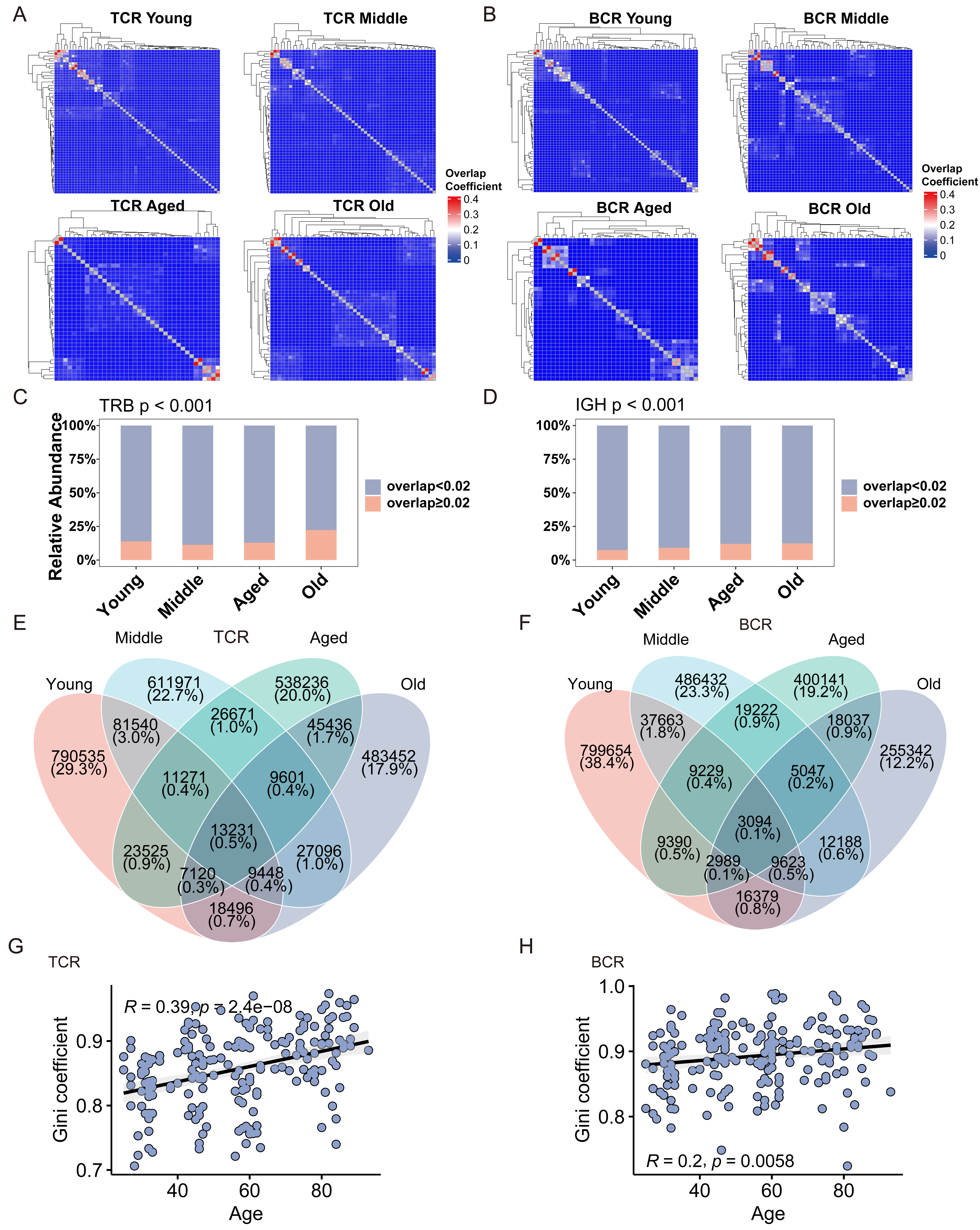 |
| --- |

**Figure S8.** **Clone and diversity analysis.**

**A–B,** Clone overlap coefficients between samples in different age groups. **C–D,** Comparison of overlap coefficients in different age groups. **E–F,** Intersections of TCR and BCR clones across different age groups. **G–H,** Correlations between Gini coefficient and age.

| 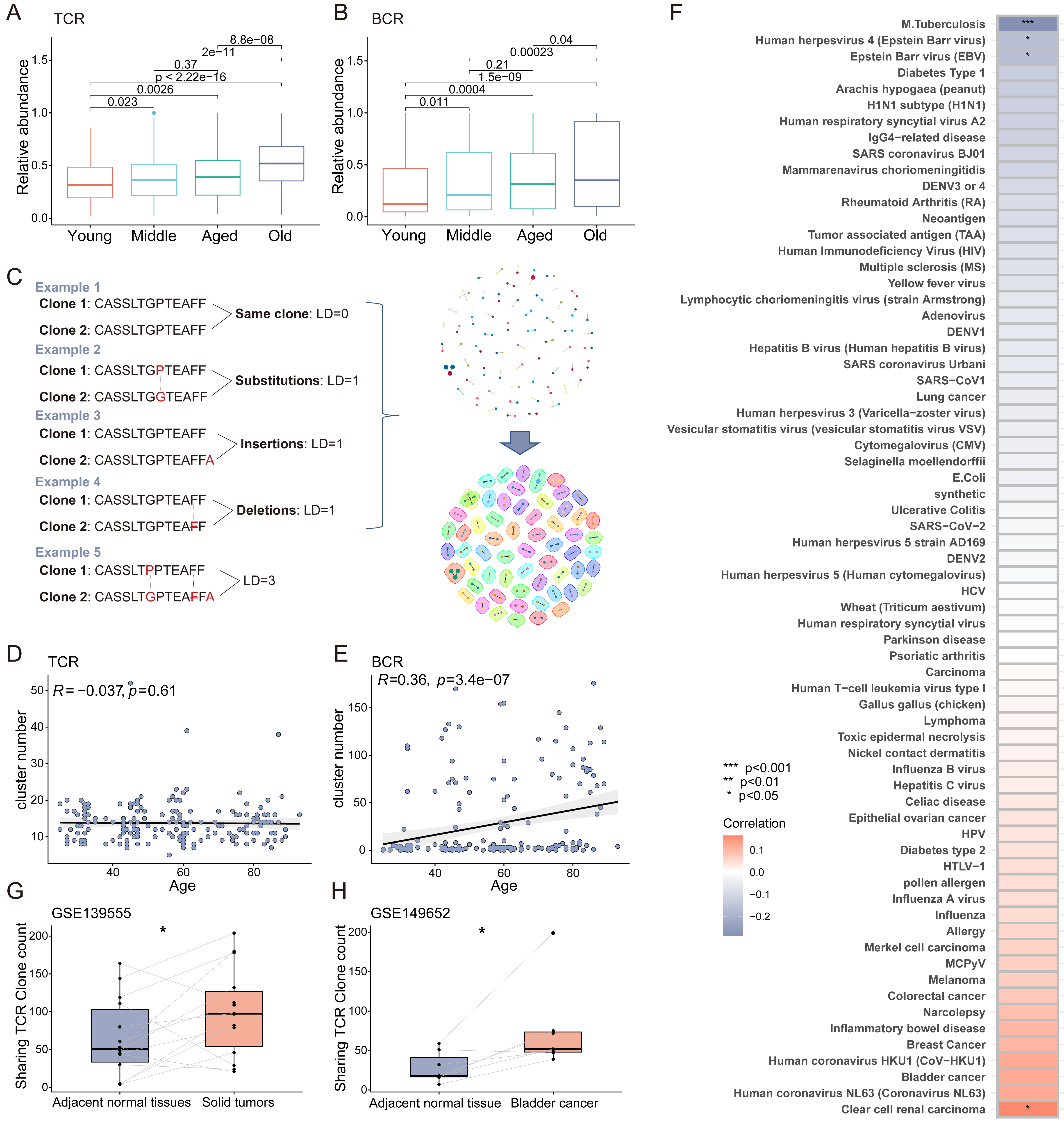 |
| --- |

**Figure S9.** **Changes in and functional analysis of the top 1000 expanded clones with age.**

**A–B,** Comparison of the total proportions of TCR and BCR clones in the top 1000 among different age groups. **C,** Schematic representation of Levenshtein distance (LD) types between clone sequences (left side), with insertions, deletions, and substitutions. The upper right network diagram shows an example of clone clustering in a sample, where each point represents a clone (colors are randomly assigned). Node size correlates with node degree (degree). Connections indicate LD ≤ 1 between clones. The lower right panel depicts the network of these clones, where clusters are circled to count of the number of clusters in the sample. **D–E,** Age-related analysis of LD similarity cluster numbers. **F,** Age-related analysis of annotation proportions of the top 1000 TCR clones. **G,** Comparison of shared clone numbers between the top 1000 peripheral blood TCR clones and various solid tumors and adjacent tissues in the GSE139555 dataset. **H,** Comparison of shared clone numbers between the top 1000 peripheral blood TCR clones and bladder cancer and adjacent tissues in the GSE149652 dataset.

| 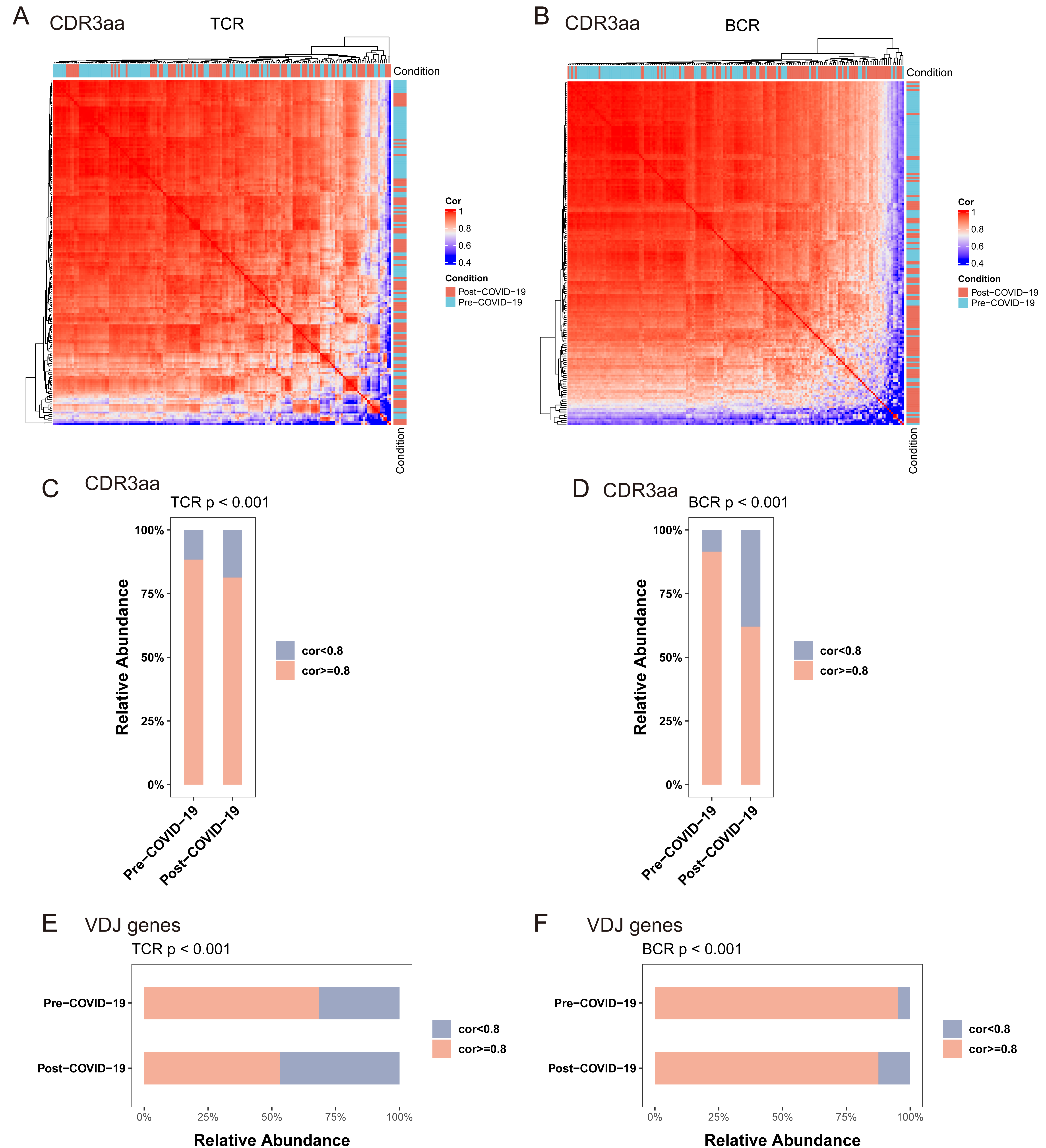 |
| --- |

**Figure S10.** **Similarity analysis of CDR3aa and VDJ gene usage rates before and after COVID-19.**

**A–D,** Comparison of the similarity of CDR3aa distributions between samples before and after COVID-19. **E–F,** Comparisons of the similarity of VDJ gene usage rates between samples before and after COVID-19.

| 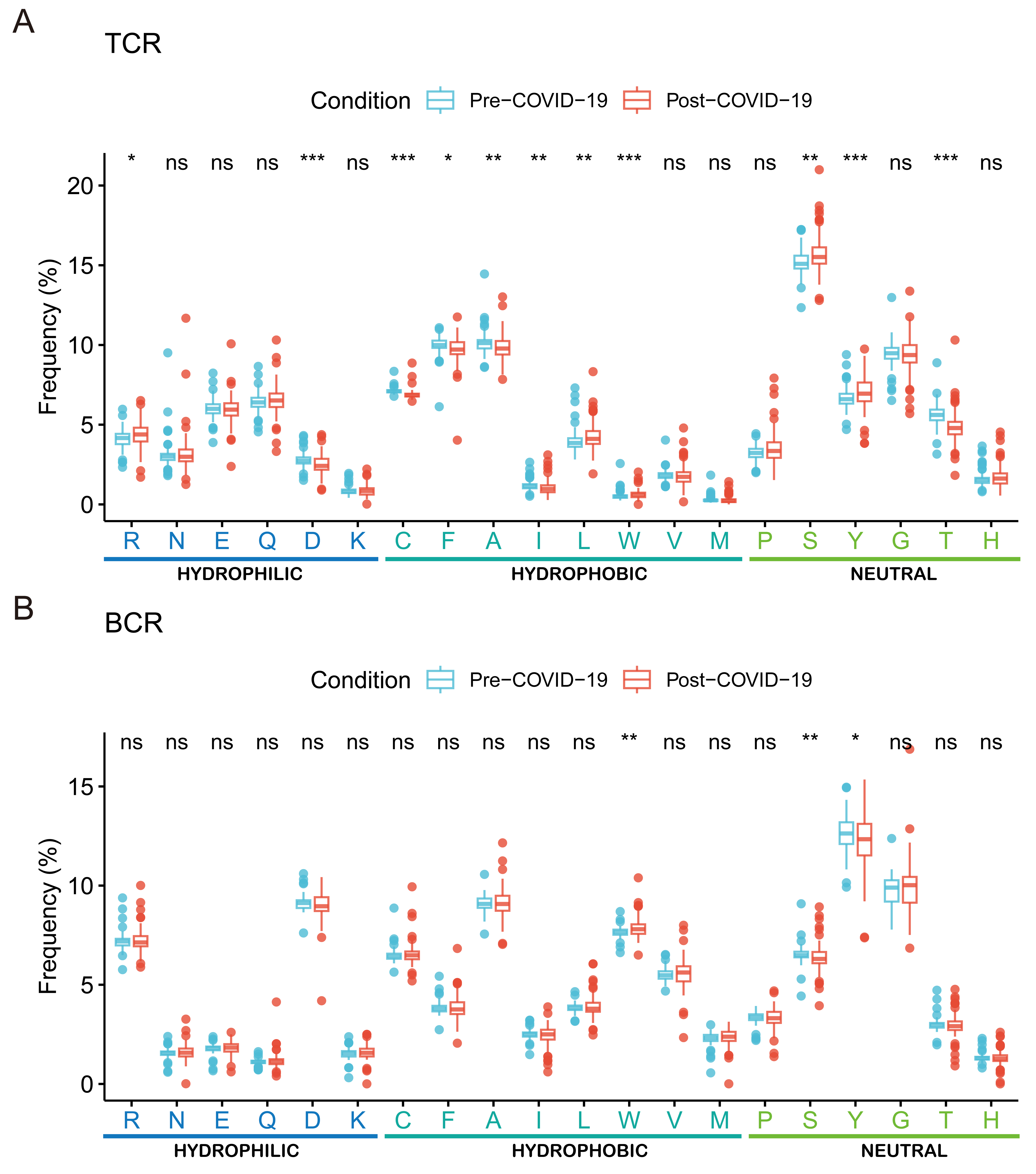 |
| --- |

**Figure S11.** **Comparison of amino acid usage rates before and after SARS-CoV-2 infection.**

**A,** TCR. **B,** BCR. **p* < 0.05, ***p* < 0.01, ****p* < 0.001, ns, no significant.

**Amino Acids Classified by Polarity:**

**1. Hydrophobic**: **A**, Alanine; **C**, Cysteine; **I**, Isoleucine; **L**, Leucine; **M**, Methionine; **F**, Phenylalanine; **W**, Tryptophan; **V**, Valine.

**2. Neutral**: **G**, Glycine; **H**, Histidine; **P**, Proline; **S**, Serine; **T**, Threonine; **Y**, Tyrosine.

**3. Hydrophilic**: **R**, Arginine; **N**, Asparagine; **D**, Aspartic acid; **Q**, Glutamine; **E**, Glutamic acid; **K**, Lysine.

| 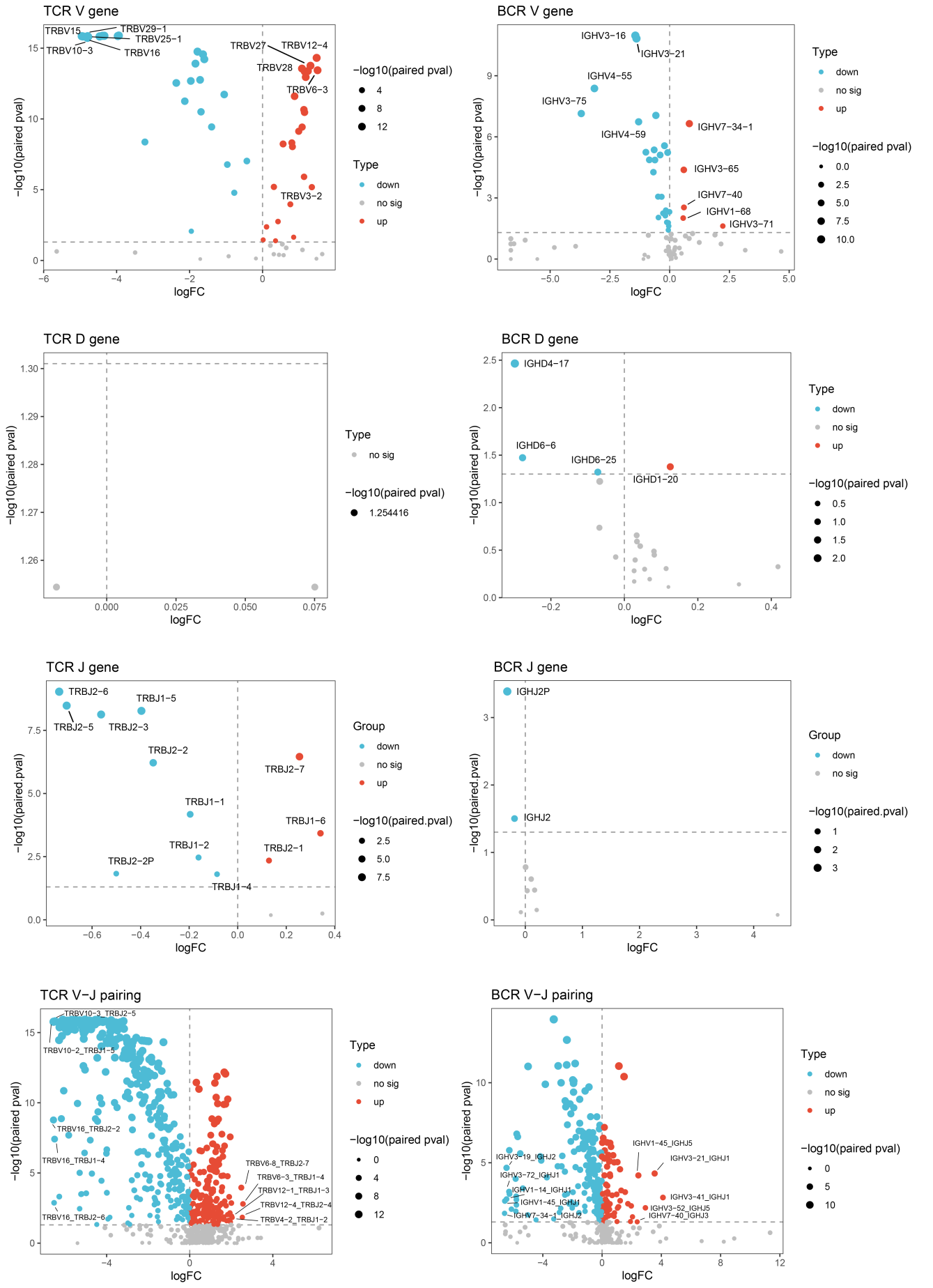 |
| --- |

**Figure S12.** **Differential analysis of gene usage before and after SARS-CoV-2 infection.**

| 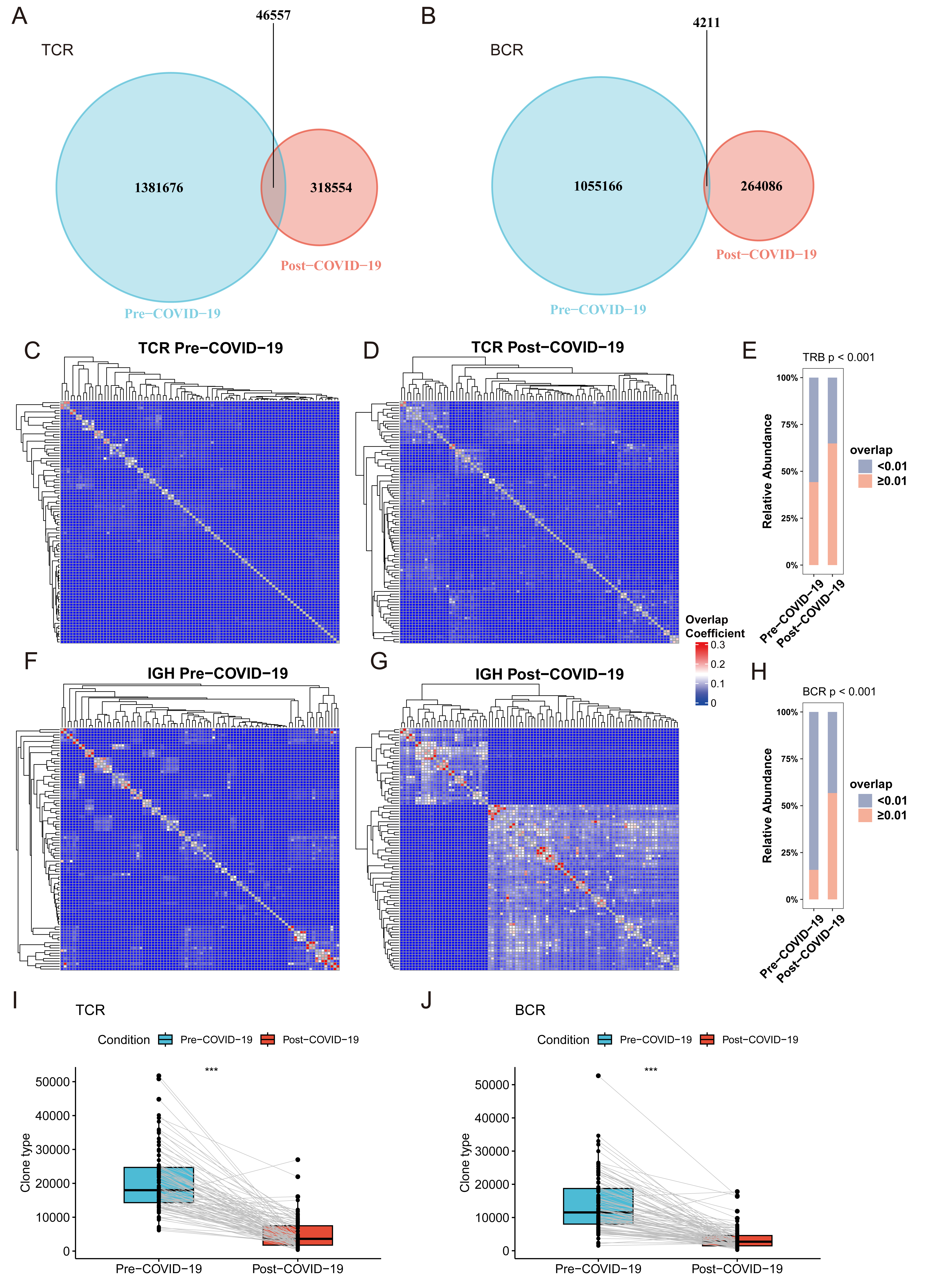 |
| --- |

**Figure S13.** **Clone analysis before and after SARS-CoV-2 infection.**

**A–B,** Intersections of all TCR (A) and BCR (B) clones in samples before and after infection. **C–H,** Analysis of TCR (C–E) and BCR (F–H) clone overlap coefficients between samples in groups before and after infection. **I–J,** Comparison of the number of TCR (I) and BCR (J) clone types before and after infection. ****p* < 0.001.

| 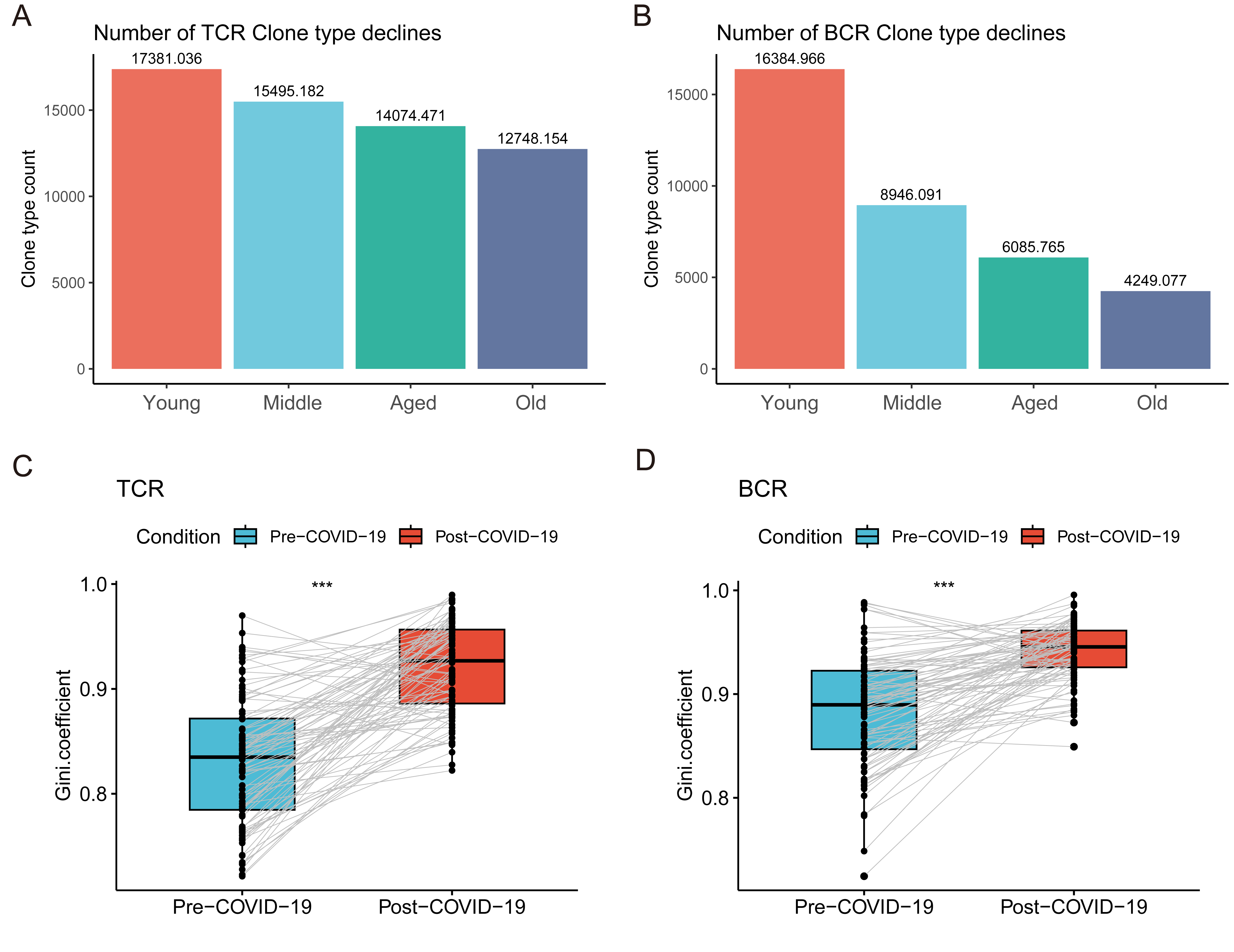 |
| --- |

**Figure S14.** **Changes in clones after SARS-CoV-2 infection.**

**A–B,** The extent of decline in TCR (A) and BCR (B) clones in different age groups after SARS-CoV-2 infection. **C–D,** Comparison of TCR (C) and BCR (D) Gini coefficient values before and after COVID-19 infection. ****p* < 0.001.

| 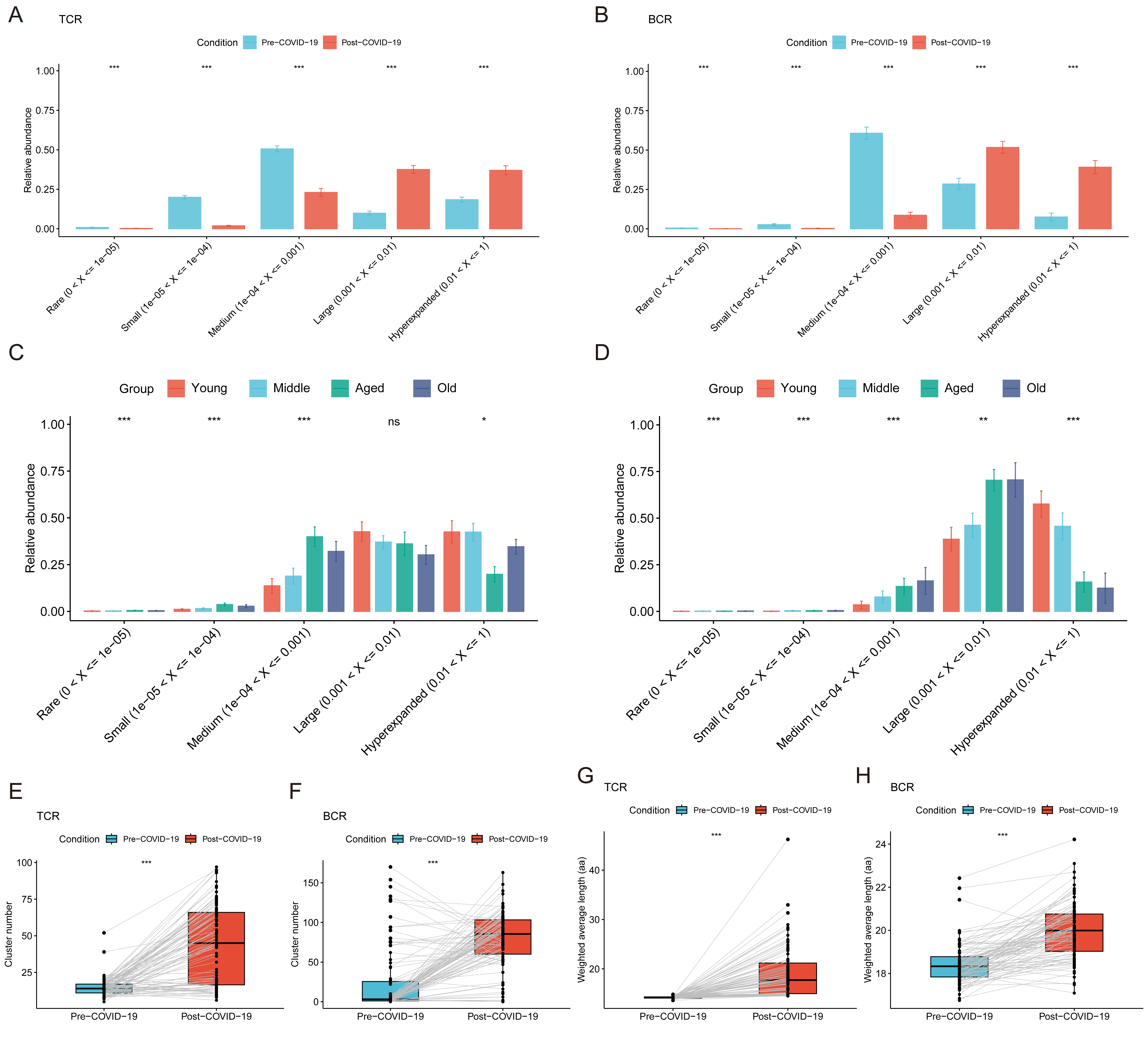 |
| --- |

**Figure S15.** **Clone and diversity analysis after COVID-19 infection.**

**A–B,** Analysis of TCR (A) and BCR (B) clone space steady state before and after COVID-19 infection. **C–D,** Analysis of TCR (C) and BCR (D) clone space steady state in patients of different age groups after COVID-19 infection. **E–F,** Cluster number statistics for the top 1000 TCR (E) and BCR (F) clones before and after infection. **G–H,** Analysis of the amino acid sequence length of the top 1000 expanded TCR (G) and BCR (H) clones. **p* < 0.05, ***p* < 0.01, ****p* < 0.001, ns, no significant.

| 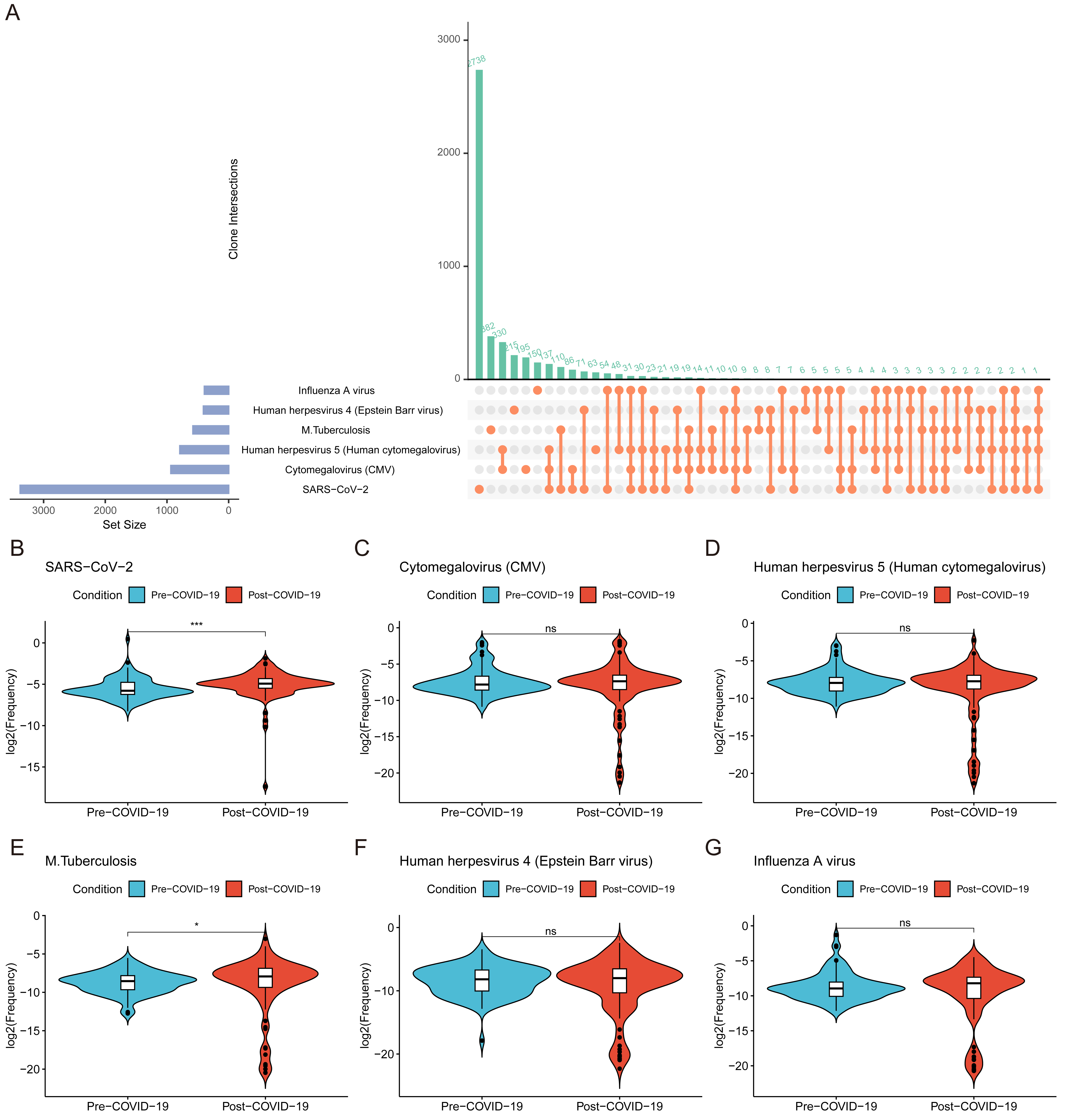 |
| --- |

**Figure S16.** **Functional analysis of the top 1000 amplified clones post-COVID-19.**

**A,** Distribution of the top 6 pathogen types successfully annotated and the number of clones for each type. **B–G,** Amplification frequency of the top 6 pathogen clonotypes before and after SARS-CoV-2 infection. **p* < 0.05, ***p* < 0.01, ****p* < 0.001, ns, no significant.

| 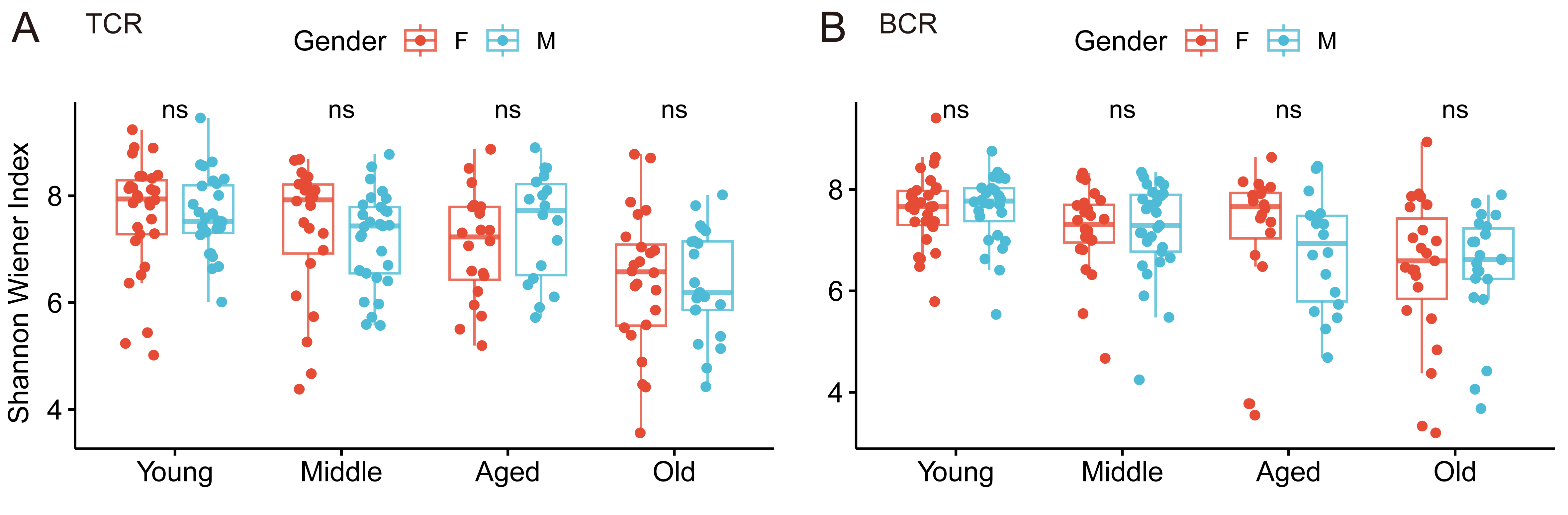 |
| --- |

**Figure S17. Comparisons of Shannon-Wiener index values between the gender.**

**A**, TCR. **B**, BCR. ns, no significant difference; F, female; M, male; ns, no significant.
